# DrABC: deep learning accurately predicts germline pathogenic mutation status in breast cancer patients based on phenotype data

**DOI:** 10.1186/s13073-022-01027-9

**Published:** 2022-02-25

**Authors:** Jiaqi Liu, Hengqiang Zhao, Yu Zheng, Lin Dong, Sen Zhao, Yukuan Huang, Shengkai Huang, Tianyi Qian, Jiali Zou, Shu Liu, Jun Li, Zihui Yan, Yalun Li, Shuo Zhang, Xin Huang, Wenyan Wang, Yiqun Li, Jie Wang, Yue Ming, Xiaoxin Li, Zeyu Xing, Ling Qin, Zhengye Zhao, Ziqi Jia, Jiaxin Li, Gang Liu, Menglu Zhang, Kexin Feng, Jiang Wu, Jianguo Zhang, Yongxin Yang, Zhihong Wu, Zhihua Liu, Jianming Ying, Xin Wang, Jianzhong Su, Xiang Wang, Nan Wu

**Affiliations:** 1 Department of Breast Surgical Oncology, National Cancer Center/National Clinical Research Center for Cancer/Cancer Hospital, Chinese Academy of Medical Sciences and Peking Union Medical College, Beijing, 100021 China; 2 Institute of Biomedical Big Data, Wenzhou Medical University, Wenzhou, 325027 China; 3 Department of Orthopedic Surgery, Peking Union Medical College Hospital, Peking Union Medical College and Chinese Academy of Medical Sciences, Beijing, 100730 China; 4 Beijing Key Laboratory for Genetic Research of Skeletal Deformity, Peking Union Medical College Hospital, Peking Union Medical College and Chinese Academy of Medical Sciences, Beijing, 100730 China; 5 Fintech Innovation Center, Southwestern University of Finance and Economics, Chengdu, 611130 China; 6 Department of Pathology, National Cancer Center /National Clinical Research Center for Cancer/Cancer Hospital, Chinese Academy of Medical Sciences and Peking Union Medical College, Beijing, 100021 China; 7 School of Biomedical Engineering, School of Ophthalmology & Optometry and Eye Hospital, Wenzhou Medical University, Wenzhou, 325027 China; 8 Department of Laboratory Medicine, National Cancer Center /National Clinical Research Center for Cancer/Cancer Hospital, Chinese Academy of Medical Sciences and Peking Union Medical College, Beijing, 100021 China; 9 Department of Breast Surgery, Guiyang Maternal and Child Healthcare Hospital, Guiyang, 550001 China; 10 Department of Breast Surgery, the Affiliated Hospital of Guizhou Medical University, Guiyang, 550004 China; 11 Department of Molecular Pathology, the Affiliated Cancer Hospital of Zhengzhou University, Zhengzhou, 450000 China; 12 Department of Breast Surgery, the Affiliated Yantai Yuhuangding Hospital of Qingdao University, Yantai, 264000 China; 13 Department of Breast Surgery, the Fourth Hospital of Hebei Medical University, Shijiazhuang, 050019 Hebei China; 14 Department of Breast Surgery, Peking Union Medical College Hospital, Peking Union Medical College and Chinese Academy of Medical Sciences, Beijing, 100730 China; 15 Department of Breast Surgery, Beijing Tiantan Hospital, Capital Medical University, Beijing, 100070 China; 16 Department of Oncology, National Cancer Center /National Clinical Research Center for Cancer/Cancer Hospital, Chinese Academy of Medical Sciences and Peking Union Medical College, Beijing, 100021 China; 17 Department of Ultrasound, National Cancer Center/National Clinical Research Center for Cancer/Cancer Hospital, Chinese Academy of Medical Sciences and Peking Union Medical College, Beijing, 100021 China; 18 PET-CT Center, National Cancer Center/National Clinical Research Center for Cancer/Cancer Hospital, Chinese Academy of Medical Sciences and Peking Union Medical College, Beijing, 100021 China; 19 Medical Research Center, Beijing Key Laboratory for Genetic Research of Skeletal Deformity & Key Laboratory of Big Data for Spinal Deformities, All at Peking Union Medical College Hospital, Peking Union Medical College and Chinese Academy of Medical Sciences, Beijing, 100730 China; 20 Department of Breast Surgical Oncology, Cancer Hospital of HuanXing, Beijing, 100021 China; 21 Key Laboratory of Big Data for Spinal Deformities, Peking Union Medical College and Chinese Academy of Medical Sciences, Beijing, 100730 China; 22 State Key Laboratory of Complex Severe and Rare Diseases, Peking Union Medical College Hospital, Peking Union Medical College and Chinese Academy of Medical Sciences, Beijing, 100730 China; 23 Machine Intelligence Group, University of Edinburgh, Edinburgh, EH8 9YL UK; 24 State Key Laboratory of Molecular Oncology, National Cancer Center/National Clinical Research Center for Cancer/Cancer Hospital, Chinese Academy of Medical Sciences and Peking Union Medical College, Beijing, 100021 China; 25 Wenzhou Institute, University of Chinese Academy of Sciences, Wenzhou, 325011 China

**Keywords:** Hereditary breast cancer, Deep learning, *BRCA1/2*, Genetic test, Genotype-phenotype correlation

## Abstract

**Background:**

Identifying breast cancer patients with DNA repair pathway-related germline pathogenic variants (GPVs) is important for effectively employing systemic treatment strategies and risk-reducing interventions. However, current criteria and risk prediction models for prioritizing genetic testing among breast cancer patients do not meet the demands of clinical practice due to insufficient accuracy.

**Methods:**

The study population comprised 3041 breast cancer patients enrolled from seven hospitals between October 2017 and 11 August 2019, who underwent germline genetic testing of 50 cancer predisposition genes (CPGs). Associations among GPVs in different CPGs and endophenotypes were evaluated using a case-control analysis. A phenotype-based GPV risk prediction model named DNA-repair Associated Breast Cancer (DrABC) was developed based on hierarchical neural network architecture and validated in an independent multicenter cohort. The predictive performance of DrABC was compared with currently used models including BRCAPRO, BOADICEA, Myriad, PENN II, and the NCCN criteria.

**Results:**

In total, 332 (11.3%) patients harbored GPVs in CPGs, including 134 (4.6%) in *BRCA2*, 131 (4.5%) in *BRCA1*, 33 (1.1%) in *PALB2*, and 37 (1.3%) in other CPGs. GPVs in CPGs were associated with distinct endophenotypes including the age at diagnosis, cancer history, family cancer history, and pathological characteristics. We developed a DrABC model to predict the risk of GPV carrier status in *BRCA1/2* and other important CPGs. In predicting GPVs in *BRCA1/2*, the performance of DrABC (AUC = 0.79 [95% CI, 0.74–0.85], sensitivity = 82.1%, specificity = 63.1% in the independent validation cohort) was better than that of previous models (AUC range = 0.57–0.70). In predicting GPVs in any CPG, DrABC (AUC = 0.74 [95% CI, 0.69–0.79], sensitivity = 83.8%, specificity = 51.3% in the independent validation cohort) was also superior to previous models in their current versions (AUC range = 0.55–0.65). After training these previous models with the Chinese-specific dataset, DrABC still outperformed all other methods except for BOADICEA, which was the only previous model with the inclusion of pathological features. The DrABC model also showed higher sensitivity and specificity than the NCCN criteria in the multi-center validation cohort (83.8% and 51.3% vs. 78.8% and 31.2%, respectively, in predicting GPVs in any CPG). The DrABC model implementation is available online at http://gifts.bio-data.cn/.

**Conclusions:**

By considering the distinct endophenotypes associated with different CPGs in breast cancer patients, a phenotype-driven prediction model based on hierarchical neural network architecture was created for identification of hereditary breast cancer. The model achieved superior performance in identifying GPV carriers among Chinese breast cancer patients.

**Supplementary Information:**

The online version contains supplementary material available at 10.1186/s13073-022-01027-9.

## Background

Breast cancer is the most common cancer in women around the world [1]. Approximately 10% of patients with breast cancer carry germline pathogenic variants (GPVs) in cancer predisposition genes (CPGs) implicated in the DNA repair pathway [2, 3]. Distinguishing breast cancer patients with GPVs is essential for employing systemic treatment strategies and risk-reducing interventions [4, 5]. However, less than 10% of these carriers are referred for genetic testing in current clinical practice due to the cost and time spent [6, 7].

The probability of carrying GPVs among breast cancer patients has long been evaluated in terms of family cancer history and clinical characteristics, such as the age at diagnosis and tumor pathological information [8, 9]. One of the most commonly used criteria is the National Comprehensive Cancer Network (NCCN) criterion [7, 10–12]. However, adhering to the current NCCN criteria would overlook nearly half of breast cancer patients with a clinically actionable GPV [7, 11–13]. Nonetheless, routine genetic testing of all or most breast cancer patients would require vastly increased genetic counseling and management, which might not be easily achieved with presently available resources [14]. Furthermore, extending population-based genetic testing to patients with low rates of or non-existent founder mutations might pose a considerable financial burden, ethical concerns, and other barriers [15, 16]. Therefore, an accurate prediction model for GPVs in clinically actionable genes is urgently needed. Recently, deep learning algorithms were demonstrated to improve clinical practice in genomic diagnostics due to their high accuracy and ability to extract information from big data [17]. Recent studies have demonstrated deep learning as a feasible and potentially useful tool for predicting germline *BRCA1/2* status for cancer patients using demographic and clinical characteristics, medical images, or pathology images [18–20]. It is not known whether deep learning algorithms can be used to improve the precise selection of breast cancer patients to undergo genetic testing.

Here, we evaluated the family history of multiple cancer types and detailed phenotypes in a multi-center cohort of 3041 female Chinese breast cancer patients who underwent multigene genetic testing. Based on the distinct endophenotypes of breast cancer patients with GPVs in genes involved in homologous recombination and other DNA repair pathways, we designed a deep learning-driven model named DrABC (DNA-repair Associated Breast Cancer) to improve the accuracy in identifying carriers for GPVs in CPGs among breast cancer patients.

## Methods

### Study participants and design

In this multi-center cohort study, we consecutively recruited unselected female patients with breast cancer from October 1, 2017, to August 31, 2019, at the Cancer Hospital of Chinese Academy of Medical Sciences and Peking Union Medical College (CHCAMS, i.e., the discovery cohort) and other six hospitals(i.e., the validation cohort), including (1) Huanxing Cancer Hospital, (2) Guiyang Maternal and Child Healthcare Hospital in Guiyang, (3) the Affiliated Cancer Hospital of Zhengzhou University, (4) the Affiliated Yantai Yuhuangding Hospital of Qingdao University, (5) the Fourth Hospital of Hebei Medical University, and (6) Beijing Tiantan Hospital all in China. The diagnosis of each patient was based on pathological results from resection specimens. This study was reviewed and approved by the ethics committees at each participating hospital. Written informed consent was obtained from each participant. This article follows the Strengthening the Reporting of Observational Studies in Epidemiology (STROBE) reporting guidelines [21].

As a result, 3041 women with breast cancer were enrolled, while 113 patients without available samples were excluded. The germline genetic test and analysis of 50 CPGs and detailed phenotypic evaluation were conducted in the remaining 2928 patients.

### Phenotype data

We collected phenotypic data including the age at diagnosis, family cancer history, personal cancer history, pathological features, molecular subtype, and clinical stage (Additional file 1: Supplementary method). Molecular subtyping was performed based on hormone receptor (HR, including estrogen receptor [ER] and progesterone receptor [PR]) and HER2 status [22]. Staging was determined according to the 8th edition of the classification of breast cancer staging from the American Joint Commission of Cancer [23].

### GPV analysis

Genomic DNA was extracted from peripheral blood or saliva. GPVs in patients from each center were analyzed by their local diagnostic laboratory, which generated a clinical genetic test report for each participant. Each laboratory provided results by the enrichment of the coding regions and consensus splice sites of 50 CPGs in the DNA repair pathway using a targeted panel followed by sequencing (Additional file 1: Supplementary method) [24, 25]. Only novel variants or variants with < 0.1% population frequency in the 1000 Genomes (October 2013) and the genome Aggregation Database (gnomAD, http://gnomad.broadinstitute.org/) were collected in this study. The clinical significance of each GPV was evaluated based on a 5-tier classification system of pathogenic/likely pathogenic (P/LP), benign/likely benign (B/LB), and variants of uncertain significance (VUS) according to guidelines of the American College of Medical Genetics and Genomics and the Association for Molecular Pathology and in-house pipeline [25–28]. The variants in *BRCA1/2* were further analyzed according to the ENIGMA expert panel review [29, 30]. For those variants without available expert panel results, the consensus classifications in ClinVar were referred to. Variants classified as P/LP were considered pathogenic in this study (Additional file 1: Supplementary method).

### DrABC model development

The DrABC risk prediction model was designed based on a hierarchical neural network that starts with an input layer of 25 neurons corresponding to features of carriers of GPVs in CPGs followed by two hidden layers. A dropout operator is applied to the hidden layers with a 25% chance of disabling a random neuron, which prevents the model from overfitting. In addition, a non-linear activation function, Scaled Exponential Linear Unit [31], is attached to the output of the hidden layers, which helps keep the representation distributions close to Gaussian. Finally, the output layer consists of two neurons with a sigmoid activation function, such that it produces two valid probabilities (i.e., in the range of [0, 1]): *P*_1_ and *P*_2_.Using *P*_1_ and *P*_2_, the final prediction is calculated using the following equations:

1 $${P}_a={P}_1,$$

2 $${P}_b={P}_1{P}_2,$$

3 $${P}_c={P}_1\left(1-{P}_2\right),$$

where *P*_*a*_ is the probability of having mutation in any CPGs, *P*_*b*_ is the probability of having *BRCA1/2* mutation, and *P*_*c*_ is the probability of having mutations in other CPGs.

With the paired input features and ground truth annotations of [*P*_*a*_, *P*_*b*_, *P*_*c*_] (in the form of one-hot encoding), we trained 101 deep learning models using cross-entropy loss via gradient descent. The final prediction is derived by aggregating results from all deep learning models through the ensemble learning strategy (Additional file 1: Supplementary method) [32, 33]. The cutoff points for each prediction scenario were determined to achieve 90% sensitivity (or the maximum sensitivity).

To evaluate the performance between the DrABC model and other machine learning models, we compared six kinds of commonly used machine learning algorithms, including a fixed grid of Generalized Linear Models (GLMs), a naive Bayes (NB) classifier, five pre-specified Gradient Boosting Machine (GBM) models, three pre-specified and a random grid of eXtreme Gradient Boosting (XGBoost) models, a default Random Forest (RF), a near-default Deep Neural Net (DNN), and a random grid of DNNs. All models were trained on the discovery dataset to predict whether a breast cancer patient carries germline pathogenic variants in any cancer predisposition genes (CPGs) using an inner five-fold cross-validation strategy. For each algorithm family, only the best model was retained to represent the maximum performance of each kind. These common machine learning algorithms were performed using the R package h2o [34].

### Statistical analysis

Student’s *t*-tests were used to analyze age at enrollment and age at diagnosis. The prevalence of personal cancer history, family cancer history, tumor size, histological grade, ER/PR/androgen receptor (AR)/HER2 status, and lymph nodes metastasis were compared using Pearson *χ*^2^ or Fisher’s exact tests. The risk of carrying a GPV in *BRCA1/2* or CPGs was also estimated using NCCN guidelines (version 1.2020) [12], BRCAPRO (version 2.1-7) [35, 36], Myriad II [37], PENN II [38], and BOADICEA (v3) [39] models in the multi-center validation cohort. Sensitivity, specificity, accuracy, and area under the curve (AUC) with the receiver operating characteristic (ROC) were calculated to evaluate the predictive performance of DrABC, other machine learning, and previous models. The performance of two ROC curves was compared through the “DeLong’s test” [40] using the algorithm of Sun and Xu [41]. Two-sided *p* < 0.05 was considered statistically significant. Statistical analysis was performed using SPSS version 15.0 (SPSS, USA) and R statistical software, version 3.5.1. The Youden index (*J* = sensitivity + specificity − 1) was used to evaluate the balance and potential effectiveness of each model with the suggested threshold [42].

## Results

### Patient characteristics

In total, patients were diagnosed at 42.9 ± 9.1 years of age, with 1168 (39.9%, 1168/2928) having early-onset cancer (age at diagnosis ≤ 40 years [43]). There were 400 (13.7%) patients with a family history of breast cancer, 86 (2.9%) patients with bilateral breast cancer, and 96 (3.3%) patients with an additional primary cancer other than breast cancer.

### Prevalence of GPVs

**Fig. 1:**
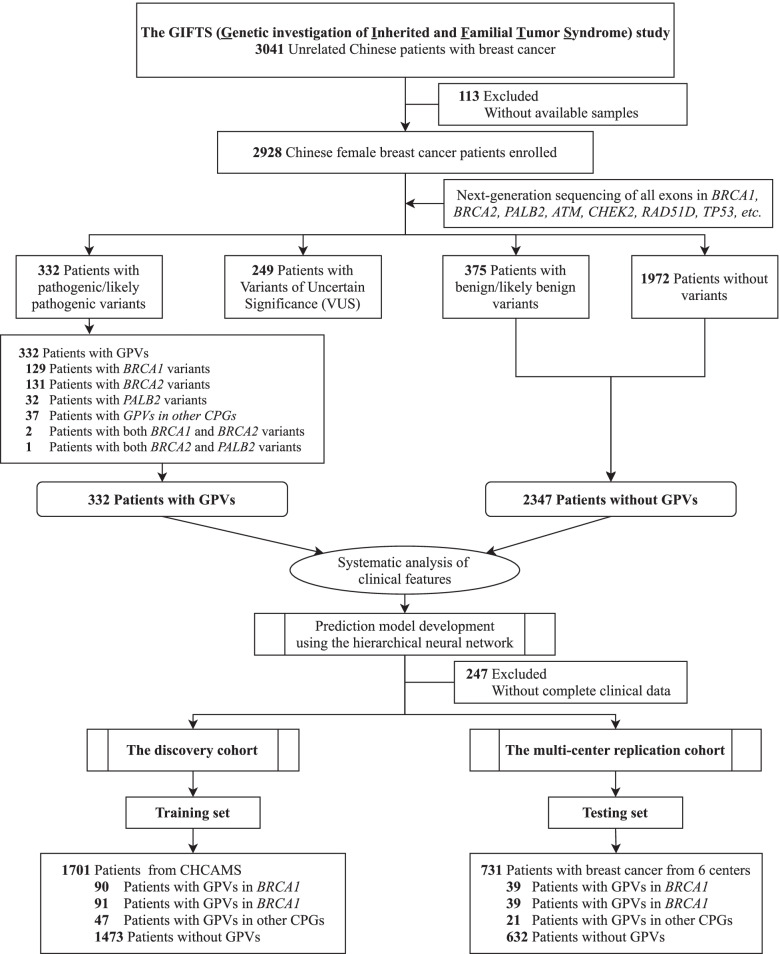
Patient enrollment and study design. GPV, germline pathogenic variant; CPG, cancer predisposition gene; CHCAMS, Cancer Hospital of Chinese Academy of Medical Sciences

**Fig. 2:**
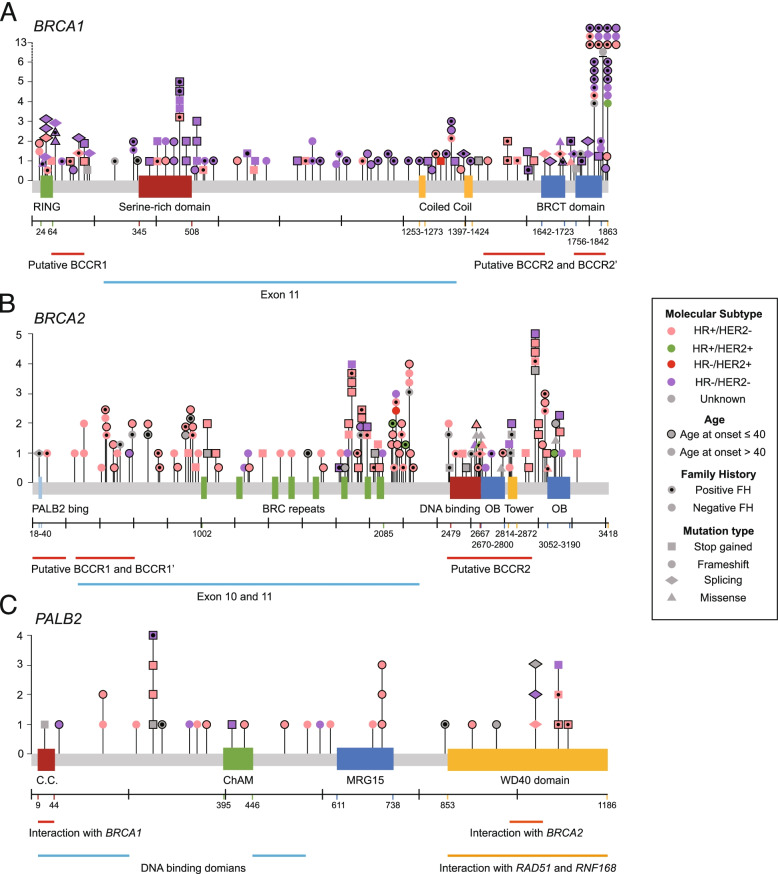
Genotype-phenotype atlas of hereditary breast cancer. **A** Germline pathogenic variants (GPVs) in *BRCA1* were found in 131 (4.5%) patients. Most *BRCA1* carriers had triple-negative breast cancer (82/131, 62.6%). **B** GPVs in *BRCA2* were found in 134 (4.6%) patients. Most *BRCA2* carriers were hormone receptor (HR)-positive and HER2-negative (90/134, 67.2%). **C** GPVs in *PALB2* were found in 33 (1.1%) patients. Most *PALB2* carriers were HR-positive and HER2-negative (21/33, 63.6%)

In total, 332 (11.3%, 332/2928) patients harbored 335 GPVs in CPGs (including 334 single nucleotide variants/indels and one deletion of *BRCA2* exons 22-24), while 295 VUS were found in 249 (8.5%) patients (Fig. 1, Additional file 2: Fig. S1 and Additional file 3: Table S1) and were excluded from further analysis to avoid potential contamination of datasets. Patients with GPVs (*n* = 332) were further divided into four subgroups according to the clinical significance of mutated genes: *BRCA1* (*n* = 131); *BRCA2* (*n* = 132) (Fig. 2A and B); other homologous recombinational repair (HRR)-related genes [44] including *PALB2* (Fig. 2C), *RAD51C*, *RAD51D*, *BARD1*, and *BRIP1* (*n* = 43); and other CPGs [45] (*n* = 26)*.*

### Association of GPVs with clinical characteristics

Compared with non-carriers, patients with GPVs in any CPGs are associated with younger onset ages (40.15 ± 8.29 in any CPGs vs. 43.43 ± 9.08 in non-carriers, *p* = 5.7 × 10^− 10^; Table 1 and Fig. 1). Furthermore, patients with GPVs in *BRCA1/2* are associated with even younger-onset ages, personal history of all cancers, previous breast cancer, and ovarian cancer, family history of breast cancer (41.2% in *BRCA1* carriers and 32.6% in *BRCA2* carriers vs. 10.8% for non-carriers, *p* = 8.4 × 10^− 18^ and 9.5 × 10^− 11^, respectively) and all cancers (64.9% in *BRCA1* carriers and 53.8% in *BRCA2* carriers vs. 30.9% for non-carriers, *p* = 1.6 × 10^− 14^ and 1.5 × 10^− 7^; Additional file 4: Fig. S2), and bilateral breast cancer (11.45% in *BRCA1* and 8.33% in *BRCA2* vs. 2.22% in non-carriers, *p* = 1.0 × 10^− 6^ and 3.6 × 10^− 4^, respectively).

**Table 1:** Comparison of clinical characteristics between patients with and without DNA-repair pathway gene mutation

Clinical characteristics	Without GPVs (***n*** = 2347)	All CPGs (***n*** = 332)	***BRCA1*** carriers (***n*** = 131)	***BRCA2*** carriers (***n*** = 132)	Other HRR-related genes (***n*** = 43)	Other CPGs (***n*** = 26)	***P*** 1	***P*** 2	***P*** 3	***P*** 4	***P*** 5
Age at enrollment ^a^	45.2 ± 8.8	42.1 ± 8.4	40.7 ± 8.6	43.1 ± 7.9	44.0 ± 9.1	41.2 ± 7.7	**2.3 × 10**^**−9**^	**1.2 × 10**^**−8**^	**7.4 × 10**^**−4**^	0.38	**0.02**
Age of onset ^a^	43.4 ± 9.1	40.2 ± 8.3	39.1 ± 8.4	40.8 ± 8.0	41.2 ± 9.1	40.5 ± 7.6	**5.7 × 10**^**−10**^	**8.6 × 10**^**−8**^	**1.2 × 10**^**−3**^	0.11	0.11
≤40 years ^b^	885 (37.7%)	186 (56.0%)	79 (60.3%)	67 (50.8%)	25 (58.1%)	15 (57.7%)	**3.5 × 10**^**−10**^	**4.6 × 10**^**−7**^	**3.2 × 10**^**−3**^	**0.01**	**0.04**
> 40 years ^b^	1462 (62.3%)	146 (44.0%)	52 (39.7%)	65 (49.2%)	18 (41.9%)	11 (42.3%)					
Personal history ^b^											
Any cancer	113 (4.8%)	39 (11.8%)	16 (12.2%)	18 (13.6%)	3 (7.0%)	2 (7.7%)	**5 × 10**^**−6**^	**9.5 × 10**^**−4**^	**1.3 × 10**^**−4**^	0.46	0.36
Previous breast cancer	47 (2.0%)	26 (7.8%)	14 (10.7%)	10 (7.6%)	1 (2.3%)	1 (3.9%)	**1.8 × 10**^**−7**^	**2.0 × 10**^**−6**^	**6.6 × 10**^**−4**^	0.59	0.41
Ovarian cancer	8 (0.3%)	4 (1.2%)	1 (0.8%)	3 (2.3%)	0 (0%)	0 (0%)	0.05	0.39	**0.02**	1	1
Family history ^b^											
Any cancer	726 (30.9%)	183 (55.1%)	85 (64.9%)	71 (53.8%)	15 (34.9%)	12 (46.2%)	**3.4 × 10**^**−17**^	**1.6 × 10**^**−14**^	**1.5 × 10**^**−7**^	0.62	0.13
Breast cancer	254 (10.8%)	108 (32.5%)	54 (41.2%)	43 (32.6%)	7 (16.3%)	4 (15.4%)	**3.2 × 10**^**−22**^	**8.4 × 10**^**−18**^	**9.5 × 10**^**−11**^	0.32	0.52
Ovarian cancer	18 (0.8%)	21 (6.3%)	19 (14.5%)	1 (0.8%)	0 (0%)	1 (3.9%)	**3.8 × 10**^**−10**^	**1.2 × 10**^**−15**^	1	1	0.19
Pancreas cancer	34 (1.5%)	11 (3.3%)	4 (3.1%)	2 (1.5%)	4 (9.3%)	1 (3.9%)	**0.02**	0.14	0.72	**4.3 × 10**^**−3**^	0.32
Prostate cancer	10 (0.4%)	0 (0%)	0 (0%)	0 (0%)	0 (0%)	0 (0%)	0.62	1	1	1	1
Esophageal cancer	82 (3.5%)	13 (3.9%)	7 (5.3%)	3 (2.3%)	2 (4.7%)	1 (3.9%)	0.64	0.23	0.62	0.66	0.61
Laryngeal cancer	12 (0.5%)	5 (1.5%)	2 (1.5%)	2 (1.5%)	0 (0%)	1 (3.9%)	0.05	0.17	0.17	1	0.13
Leukemia	13 (0.6%)	5 (1.5%)	1 (0.8%)	3 (2.3%)	0 (0%)	1 (3.9%)	0.06	0.53	0.05	1	0.14
Male breast cancer	2 (0.1%)	4 (1.2%)	0 (0%)	4 (3.0%)	0 (0%)	0 (0%)	**2.8 × 10**^**−**3^	1	**1.1 × 10**^**−4**^	1	1
Tumor size ^b^											
≤ 2 cm	1099 (46.8%)	146 (44.0%)	51 (38.9%)	62 (47.0%)	18 (41.9%)	15 (57.7%)	0.16	0.06	0.63	0.41	0.28
> 2 cm	899 (38.3%)	143 (43.1%)	60 (45.8%)	56 (42.4%)	20 (46.5%)	7 (27.0%)					
Histology ^b^											
IDC	1825 (77.8%)	295 (88.9%)	122 (93.1%)	114 (86.4%)	38 (88.4%)	21 (80.8%)	**1 × 10**^**−6**^	**5 × 10**^**−6**^	**0.02**	0.14	0.82
DCIS	184 (7.8%)	11 (3.3%)	0 (0%)	7 (5.3%)	2 (4.7%)	2 (7.7%)	**2.1 × 10**^**−3**^	**4.9 × 10**^**−5**^	0.40	0.77	1
Lobular	45 (1.9%)	6 (1.8%)	1 (0.8%)	3 (2.3%)	1 (2.3%)	1 (3.9%)	1	0.51	0.74	0.57	0.40
Mucinous	45 (1.9%)	3 (0.9%)	0 (0%)	2 (1.5%)	1 (2.3%)	0 (0%)	0.27	0.17	1	0.57	1
Medullary	11 (0.5%)	4 (1.2%)	2 (1.5%)	2 (1.5%)	0 (0%)	0 (0%)	0.10	0.15	0.15	1	1
Other ^c^	34 (1.5%)	4 (1.2%)	2 (1.5%)	1 (0.8%)	0 (0%)	1 (3.9%)	1	0.72	1	1	0.32
Grade ^b^											
I	139 (5.9%)	2 (0.6%)	0 (0%)	2 (1.5%)	0 (0%)	0 (0%)	**2 × 10**^**−6**^	**6.8 × 10**^**−4**^	**0.03**	0.18	0.40
II	1004 (42.8%)	129 (38.9%)	26 (19.9%)	67 (50.8%)	26 (60.5%)	10 (38.5%)	0.19	**8.4 × 10**^**−8**^	0.09	**0.03**	0.70
III	587 (25.0%)	134 (40.4%)	85 (64.9%)	35 (26.5%)	8 (18.6%)	6 (23.1%)	**1.5 × 10**^**−8**^	**1.5 × 10**^**−20**^	0.68	0.38	1
ER status ^b^											
Positive	1592 (67.8%)	190 (57.2%)	34 (26.0%)	107 (81.1%)	30 (69.8%)	19 (73.1%)	**1 × 10**^**−6**^	**4.1 × 10**^**−25**^	**0.01**	0.86	0.82
Negative	612 (26.1%)	134 (40.4%)	94 (71.8%)	22 (16.7%)	12 (27.9%)	6 (23.1%)					
PR status ^b^											
Positive	1571 (66.9%)	192 (57.8%)	35 (26.7%)	107 (81.1%)	32 (74.4%)	18 (69.2%)	**2.3 × 10**^**−5**^	**2.6 × 10**^**−23**^	**2.3 × 10**^**−3**^	0.61	1
Negative	631 (26.9%)	131 (39.5%)	93 (71.0%)	21 (15.9%)	10 (23.3%)	7 (26.9%)					
AR status ^b^											
Positive	710 (30.3%)	69 (20. 8%)	15 (11.5%)	33 (25.0%)	10 (23.3%)	11 (42.3%)	**2.8 × 10**^**−14**^	**4.6 × 10**^**−21**^	0.19	**0.02**	1
Negative	175 (7.5%)	73 (22.0%)	50 (38.2%)	13 (9.9%)	8 (18.6%)	2 (7.7%)					
HER2 status ^b^											
Positive	507 (21.6%)	15 (4.5%)	2 (1.5%)	6 (4.6%)	0 (0%)	7 (26.9%)	**8.1 × 10**^**−17**^	**4.2 × 10**^**−11**^	**1.1 × 10**^**−7**^	**4.3 × 10**^**−5**^	0.48
Negative	1317 (56.1%)	277 (83.4%)	120 (91.6%)	103 (78.0%)	37 (86.1%)	17 (65.4%)	**2.8 × 10**^**−23**^	**3.7 × 10**^**−18**^	**4.5 × 10**^**−7**^	**6.3 × 10**^**−5**^	0.43
Uncertain	342 (14.6%)	27 (8.1%)	3 (2.3%)	18 (13.6%)	4 (9.3%)	2 (7.7%)	**1.2 × 10**^**−3**^	**6 × 10**^**−6**^	0.90	0.51	0.57
TNBC ^b^	303 (12.9%)	109 (32.8%)	82 (62.6%)	15 (11.4%)	8 (18.6%)	4 (15.4%)	**9.5 × 10**^**−18**^	**6.8 × 10**^**−37**^	0.69	0.25	0.77
Ki67 ^b^											
≤ 30%	1254 (53.4%)	134 (40.4%)	21 (16.0%)	74 (56.1%)	23 (53.5%)	16 (61.5%)	**1.0 × 10**^**−7**^	**1.0 × 10**^**−19**^	0.85	0.87	0.54
> 30%	852 (36.3%)	175 (52.7%)	98 (74.8%)	52 (39.4%)	17 (39.5%)	8 (30.8%)					
EGFR ^b^											
Positive	467 (19.9%)	110 (33.1%)	72 (55.0%)	23 (17.4%)	9 (20.9%)	6 (23.1%)	**2.5 × 10**^**−5**^	**2.4 × 10**^**−17**^	0.11	0.85	0.79
Negative	1026 (43.7%)	132 (39.8%)	24 (18.3%)	75 (56.8%)	22 (51.2%)	11 (42.3%)					
CK5/6 ^b^											
Positive	309 (13.2%)	90 (27.1%)	61 (46.6%)	16 (12.1%)	10 (23.3%)	3 (11.5%)	**9.0 × 10**^**−8**^	**1.1 × 10**^**−17**^	0.51	0.19	0.78
Negative	1382 (58.9%)	181 (54.5%)	46 (35.1%)	89 (67.4%)	26 (60.5%)	20 (76.9%)					
P53 ^b^											
Gain-of-function	605 (25.8%)	100 (30.1%)	46 (35.1%)	39 (29.6%)	10 (23.3%)	5 (19.2%)	0.10	**0.02**	0.36	0.86	0.65
Loss-of-function	227 (9.7%)	49 (14.8%)	33 (25.2%)	6 (4.6%)	4 (9.3%)	6 (23.1%)	**6.7 × 10**^**−3**^	**9.5 × 10**^**−7**^	0.05	1	**0.04**
Wildtype	629 (26.8%)	103 (31.0%)	21 (16.0%)	55 (41. 7%)	20 (46.5%)	7 (26.9%)	0.11	**5.7 × 10**^**−3**^	**4.1 × 10**^**−4**^	**0.01**	1
Bilateral breast cancer ^b^	52 (2.2%)	28 (8.4%)	15 (11.5%)	11 (8.3%)	1 (2.3%)	1 (3.9%)	**9.2 × 10**^**−8**^	**1.0 × 10**^**−6**^	**3.7 × 10**^**−4**^	0.62	0.45
Lymph nodes status ^b^											
Positive	892 (38.0%)	153 (46.1%)	43 (32.8%)	75 (56.8%)	21 (48.8%)	14 (53.9%)	**0.01**	0.13	**4 × 10**^**−6**^	0.13	0.05
Negative	1151 (49.0%)	143 (43.1%)	76 (58.0%)	44 (33.3%)	16 (37.2%)	7 (26.9%)					

^a^Mean ± SD, year, Student’s *T* test

^b^No. (%), Pearson’s chi-square test or Fisher’s exact test

^c^Others include metaplastic cancer, sieve cancer, Paget’s disease, micropapillary cancer, secretory cancer, tubule cancer

^d^*P* < 0.05 is considered significant. P1 non-carriers vs. all CPGs carriers, P2 non-carriers vs. *BRCA1* carriers, P3 non-carriers vs. *BRCA2* carriers, P4 non-carriers vs. other HRR-related genes carriers, P5 non-carriers vs. other CPGs carriers

^e^Numbers of patients with each unknown characteristic were not shown

*Abbreviation*: *GPV* germline pathogenic variant, *CPG* cancer predisposition gene, *HRR* homologous recombinational repair

In particular, patients with GPVs in *BRCA1* are associated with a family history of ovarian cancer (14.5% for *BRCA1* carriers vs. 0.8% for non-carriers, *p* = 1.2 × 10^− 15^), more grade III (64.89% for *BRCA1* carriers vs. 25.01% for non-carriers, *p* = 1.5 × 10^− 20^), more negative cases in ER, PR, and AR (71.76%, 70.99%, and 38.17% for *BRCA1* carriers vs. 26.08%, 26.89%, and 7.46% for non-carriers, *p* = 4.1 × 10^− 25^, 2.6 × 10^− 23^, and 4.6 × 10^− 21^, respectively). Significantly more breast cancer with ki67 > 30%, EGFR-positive breast cancer, and CK5/6-positive breast cancers were also seen in *BRCA1* mutation carriers (Table 1). Meanwhile, patients with GPVs in *BRCA2* are associated with a family history of leukemia or male breast cancer, lymph node metastasis (56.82% in *BRCA2* carriers vs. 38.01% in non-carriers, *p* = 4.0 × 10^− 5^), more positive cases in ER and PR (81.06% and 81.06% for *BRCA2* carriers vs. 67.83% and 66.94%for non-carriers, *p* = 7.8 × 10^− 3^ and 2.3 × 10^− 3^, respectively), and more wild-type P53 (41.7% for *BRCA2* carriers vs. 26.8% for non-carriers, *p* = 4.1 × 10^− 4^). Besides, patients with GPVs in other HRR-related genes are associated with a family history of pancreas cancer (9.3% in other HRR-related genes carriers vs. 1.5% in non-carriers, *p* = 4.3 × 10^− 3^) and more wild-type P53, while patients with GPVs in other CPGs are also associated with lymph node metastasis (Table 1).

However, HER2-positive status was less common among patients with HRR-related GPVs but not among those with GPVs in other CPGs (1.5% for *BRCA1* carriers, 4.6% for *BRCA2* carriers, 0% for other HRR-related gene carriers, 26.9% for other CPG carriers, vs. 21.6% for non-carriers, *p* = 4.2 × 10^− 11^, 1.1 × 10^− 7^, 4.3 × 10^− 5^, and 0.48, respectively). Triple-negative breast cancer was more common among patients with GPVs in *BRCA1* than among non-carriers (62.6% vs. 12.9%, *p* = 6.8 × 10^− 37^; Table 1). However, most *BRCA2* and other HRR-related gene mutation carriers were HR+/HER2− (66.67% and 67.44%, respectively; Additional file 5: Fig. S3). When combining molecular subtypes with the age at diagnosis and family cancer history, the CPG mutation carriers were further enriched accordingly (Additional file 6: Fig. S4).

### Using a deep learning model to predict GPVs in DNA repair genes

To ensure data integrity and cleanness, 249 patients with VUSs and 247 patients without complete clinical information or family cancer history were excluded from model construction [46]. A total of 1701 patients from the CHCAMS constituted the discovery cohort, and 731 patients from six other institutions constituted the independent multi-center validation cohort (Additional file 7: Fig. S5).

We used 25 clinical features associated with GPVs in CPGs to develop the prediction model. These 25 features correspond to an input layer of 25 neurons (Additional file 8: Table S2), followed by two hidden layers of 16 and 8 neurons, respectively (Additional file 9: Fig. S6). As a result, DrABC achieved a superior performance through the inner five-fold cross-validation in the discovery cohort, which was slightly higher than other traditional machine learning models but without significance (*p* > 0.05 when comparing each model with the DrABC; Additional file 10: Fig. S7).

### Performance of DrABC versus previous models

**Fig. 3:**
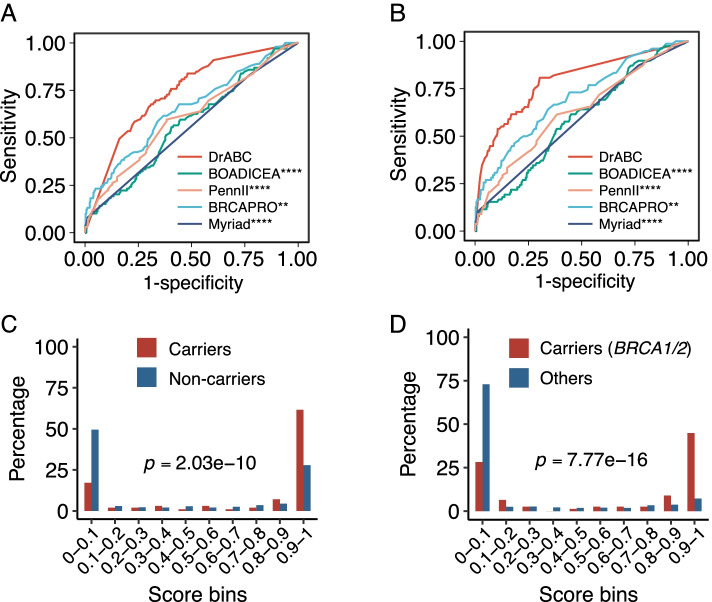
Performance of risk prediction models for hereditary breast cancer. **A** DrABC performed better than previous models in predicting germline pathogenic variants (GPVs) in any cancer predisposition genes (CPGs) (AUCs of 0.74 for DrABC, 0.65 for BRCAPRO, 0.57 for BOADICEA, 0.56 for Myriad, and 0.61 for PENNII). **B** In predicting GPVs in *BRCA1/2*, the AUC of DrABC was 0.79 (95% CI, 0.74–0.85) for the validation cohort, which was superior to those for previous models (0.70 for BRCAPRO, 0.59 for BOADICEA, 0.59 for Myriad, and 0.63 for PENN II). **C**, **D** The probabilities generated by DrABC were distributed differently between non-carriers and patients with GPVs in any CPG **(C)** or *BRCA1/2* **(D)**. **p < 0.01, ****p < 0.0001, when comparing with the DrABC

DrABC generates probabilities of whether a breast cancer patient carries GPVs in *BRCA1/2*, other CPGs except for *BRCA1/2*, or any CPG. In predicting GPVs in any CPG, the AUCs for DrABC were 0.80 (95% CI, 0.78–0.83) for the discovery cohort and 0.74 (95% CI, 0.69–0.79) for the validation cohort, which were superior to those for previous models (AUC = 0.65 for BRCAPRO [35], AUC = 0.57 for BOADICEA [39], AUC = 0.56 for Myriad [37], and AUC = 0.61 for PENN II [38] in the validation cohort; *p* < 0.01 when comparing each model with the DrABC; Fig. 3A, Table 2, and Additional file 11: Table S3). Of the 731 patients in the multi-center validation cohort, 513 (70.2%) met NCCN criteria for genetic testing criteria and 218 (29.8%) did not. Patients meeting NCCN criteria were more likely to carry GPVs in any CPG than patients not meeting the criteria (15.2% [78/513] vs. 9.6% [21/218], *p* = 0.045; OR = 1.7 [95% CI, 1.0–2.8)]. As a result, the NCCN criteria showed a sensitivity of 78.8%, specificity of 31.2%, and accuracy of 37.6% (Table 2). Expansion of NCCN criteria [13] to include all patients diagnosed with breast cancer at ≤ 65 years of age could increase the sensitivity to 100% but reduced specificity to 2.5% and accuracy to 15.7%. When achieving the highest detection rate, DrABC had a sensitivity of 90.8% and specificity of 53.2% for all GPVs in the discovery cohort and a sensitivity of 83.8% and specificity of 51.3% for all GPVs in the multi-center validation cohort (Table 2 and Additional file 12: Table S4).

**Table 2:** The prediction accuracy of the algorithms and NCCN criteria in multi-center validation cohort

	DrABC	BRCAPRO^**a**^	BOADICEA^**a**^	Myriad^**a**^	PENNII^**b**^	NCCN	NCCN expansion^**c**^
*BRCA1/2*							
AUC (95%CI)	0.792 (0.735–0.848)	0.699 (0.635–0.763)	0.586 (0.521–0.651)	0.587 (0.537–0.637)	0.628 (0.560–0.697)	NA	NA
Sensitivity	82.1%	53.8%	15.4%	9.0%	61.5%	83.3%	100%
Specificity	63.1%	72.1%	90.2%	98.9%	61.6%	31.4%	2.5%
Youden Index^d^	45.2%	25.9%	5.6%	7.9%	23.1%	14.7%	2.5%
All cancer predisposition genes							
AUC (95%CI)	0.737 (0.687–0.787)	0.650 (0.589–0.711)	0.571 (0.510–0.631)	0.556 (0.508–0.603)	0.606 (0.543–0.668)	NA	NA
Sensitivity	83.8%	45.5%	15.2%	7.1%	58.6%	78.8%	100%
Specificity	51.3%	71.6%	90.3%	98.9%	61.9%	31.2%	2.5%
Youden Index^d^	35.1%	17.1%	5.5%	6.0%	20.5%	10.0%	2.5%

^a^The cutoff values were set as 5%

^b^The cutoff values were set as 10%

^c^Expansion of the NCCN criteria included all women diagnosed with breast cancer younger than 65 years of age

^d^The Youden index was calculated as J = sensitivity+specificity-1

*Abbreviation*s: *AUC* area under the curve, *CI* confidence interval, *NA* not applicable

In predicting GPVs in *BRCA1/2*, the AUCs for DrABC were 0.81 (95% CI, 0.78–0.84) for the discovery cohort and 0.79 (95% CI, 0.74–0.85) for the validation cohort, which were also superior to those for previous models (AUC = 0.70 for BRCAPRO [35], AUC = 0.59 for BOADICEA [39, 47], AUC = 0.59 for Myriad [37], and AUC = 0.63 for PENN II [38] in the validation cohort; *p* < 0.01 when comparing each model with the DrABC; Fig. 3B, Table 2, and Additional file 11: Table S3). The DrABC had a sensitivity of 85.6% and specificity of 65.5% for GPVs in *BRCA1/2* in the discovery cohort and a sensitivity of 82.1% and specificity of 63.1% for GPVs in *BRCA1/2* in the validation cohort, when achieving the highest detection rate (Additional file 12: Table S4). Compared to previous models, the DrABC demonstrated the highest Youden index with the corresponding threshold for detecting GPVs in *BRCA1/2* or any CPG (Table 2), suggesting DrABC has a more balanced performance compared with previous models.

The probabilities generated by DrABC were distributed differently between non-carriers and patients with GPVs in any CPG (*p* = 2.0 × 10^− 10^; Fig. 3C) or *BRCA1/2* (*p* = 7.8 × 10^− 16^; Fig. 3D and Additional file 13: Fig. S8), suggesting its capability in distinguishing patients with hereditary breast cancer. However, DrABC was less satisfactory in predicting GPVs in other CPGs, with AUCs of 0.72 (95% CI, 0.64–0.79) in the discovery cohort and 0.58 (95% CI, 0.46–0.70) in the validation cohort, which was still higher than other models (AUC range = 0.44-0.53 in the validation cohort; Table 2 and Additional file 14: Fig. S9) but without significant difference (*p* > 0.05 when comparing each model with the DrABC; Additional file 11: Table S3). There was no significant distribution difference between non-carriers and patients with GPVs in CPGs other than *BRCA1/2* (*p* = 0.39; Additional file 14: Fig. S9).

### Contributions of family cancer history and pathological features to DrABC performance

To identify their contributions of features to the deep-learning model, we assessed the performance of DrABC after eliminating family cancer history or pathological feature data in the validation cohort. Eliminating family cancer history data did not reduce the performance of DrABC, with AUCs of 0.72 in predicting GPVs in any CPG, 0.75 in predicting GPVs in *BRCA1/2*, and 0.52 in predicting GPVs in other CPGs. However, eliminating pathological feature data reduced the performance of DrABC, with AUCs of 0.62 in predicting GPVs in any CPG, 0.66 in predicting GPVs in *BRCA1/2*, and 0.44 in predicting GPVs in other CPGs (Additional file 15: Fig. S10). Therefore, pathological feature represents an important predictive factor for hereditary breast cancer.

### Reconstructing previous prediction models using in-house data in discovery cohort

To investigate the contribution of the Chinese-specific training dataset to the superior performance of the DrABC model to the previous model, we reconstructed the previous prediction models of BRCAPRO, BOADICEA, Myriad, and PENN II using the underlying algorithms (i.e., Bayes’ theorem for BRCAPRO and BOADICEA, Logistic regression for Myriad and PENN II; through the R package h2o [34]) and input variables (Additional file 16: Table S5) [35–39] of each model. These reconstructed models were trained in the Chinese discovery cohort and validated in the multi-center validation cohort. In predicting GPVs in any CPG, DrABC was superior to the reconstructed models of BRCAPRO, Myriad, and PENN II (AUC = 0.74 for DrABC, AUC = 0.64 for BRCAPRO, AUC = 0.63 for Myriad, and AUC = 0.66 for PENN II in the validation cohort; *p* < 0.01 when comparing each model with the DrABC; Additional file 17: Fig. S11 and Additional file 18: Table S6). Similarly, in predicting GPVs in *BRCA1/2*, DrABC was superior to these three reconstructed models (AUC = 0.79 for DrABC, AUC = 0.68 for BRCAPRO, AUC = 0.68 for Myriad, and AUC = 0.70 for PENN II in the validation cohort; *p* < 0.01 when comparing each model with the DrABC; Additional file 17: Fig. S11 and Additional file 18: Table S6). However, there was no significant difference between the AUCs for DrABC and the reconstructed BOADICEA model in both predicting GPVs in any CPG and *BRCA1/2* (AUC = 0.75 and 0.78 for BOADICEA, *p* = 0.48 and 0.32 when comparing each model with the DrABC, respectively; Additional file 17: Fig. S11 and Additional file 18: Table S6).

### Online DrABC tool

We implemented a website interface (http://gifts.bio-data.cn/) to accommodate extensions to the DrABC model and make it easily accessible to healthcare providers and researchers (Additional file 19: Fig. S12). The user guide was provided in the Additional file 20: A user guide for the DrABC model.

## Discussion

Breast cancer patients with GPVs in *BRCA1/2* and other breast cancer-associated genes benefit from particular patterns of systemic treatments and risk-reducing interventions [48]. Although risk prediction models have been developed for combined groups of patients with breast or ovarian cancer as well as healthy individuals with a family history of hereditary breast and ovarian cancer [36, 37, 49, 50], no clinical tool has been specifically developed for patients already diagnosed with breast cancer. Therefore, we developed and validated a reliable prediction model using deep learning algorithms to identify GPV carriers among unselected breast cancer patients with better accuracy than previous models and no trend toward overfitting.

In this study, we have compared and tested the currently available risk prediction models and identified the shortfalls and limitations as follows: (1) the probability of carrying GPVs was derived from data from multi-generation families and computed based on the family history of specific cancers, age at diagnosis, and ancestry [37, 50, 51]. Thus, their performances in small family structures with simple pedigrees would be significantly limited [8]. (2) Evolutionarily recent or de novo mutations may have a more significant influence on disease susceptibility or protection than ancient mutations (Additional file 16: Table S5) [52]. (3) The vast majority of models were developed based on the data driven from European populations, but the performance in Asian populations has not been validated [53]. (4) Most of the existing models were specifically designed to predict the GPV carrier risk in *BRCA1*/2 genes and thus cannot be readily used to assess the risk for other breast cancer predisposition loci, which are also important for personalized healthcare decisions.

Thus, to identify whether the superior performance of DrABC may also be attributed to its Chinese-specific training dataset, we imitated the previous prediction models of BRCAPRO, BOADICEA, Myriad, and PENN II using the corresponding algorithms and input variables and trained them in the discovery cohort of this study. As a result, the performance of DrABC was superior to those of the reconstructed models of BRCAPRO, Myriad, and PENN II, but similar to the reconstructed BOADICEA model. Notably, only DrABC and the reconstructed BOADICEA model have incorporated pathological features in the algorithm. Collectively, the DrABC model has shown better performance in the Chinese population than all these previous models in their current versions. After training these previous models with the Chinese-specific dataset, the previous models without the inclusion of pathological information still cannot compete with the DrABC model, while the BOADICEA model involving the pathological features demonstrated similar performance to the DrABC model.

In comparison with traditional machine learning models, although DrABC achieved a slightly superior performance than other traditional machine learning models, there was no significant difference among them (Additional file 10: Fig. S7). While the difference between the performance of the machine learning models was also not observed in a previous study of predicting GPVs status in pancreatic cancer patients [20]. However, based on the similar deep learning technique, DNN models had the worst performance with an AUC of 0.75, suggesting that DNNs in particular are difficult to perform well without ingenious design. In addition, as a complex classification task with three categories, we specially designed the prediction model based on a hierarchical neural network, producing two probabilities: *P*_1_ and *P*_2_, where *P*_1_ is the probability of having a mutation in any CPGs, *P*_2_ is the probability of having *BRCA1/2* mutation when the patient is known to carrier mutation in any CPGs. To sum up, DrABC is a specially designed and well-performed model for this scenario.

As each CPG has distinct endophenotypes in terms of clinical and pathological features, the detailed phenotype of a proband with breast cancer should be incorporated in risk prediction. However, previously incorporating ER/PR/HER2 status into the BOADICEA model did not improve its predictive accuracy [53], inconsistent with the present study. Intriguingly, pathological features contributed more than family cancer history to the ability of DrABC to predict GPVs in *BRCA1/2* and any CPGs, which might contribute to the superior performance of DrABC and the reconstructed BOADICEA model than the other previous models.

Asian breast cancer patients exhibit several unique features. Breast cancer is diagnosed at much younger ages in Asian women than in women from Western countries [1, 54]. Moreover, *BRCA2* mutations are more common than *BRCA1* mutations in Asian women as compared with Caucasian women [55, 56]. However, we found that breast cancer patients with GPVs in *BRCA2* have less distinct endophenotypes than those with GPVs in *BRCA1*. These two features reduce the performance of previous risk prediction models and criteria [53]. To our knowledge, DrABC is the first available GPVs risk prediction model suitable for Asian breast cancer patients, which might contribute to the better performance than previous models based on Western populations.

Therefore, we introduced an applicable pipeline for GPV carrier risk assessment among patients with breast cancer (Additional file 21: Fig. S13). This approach would strike a balance between identifying more GPV carriers and testing fewer breast cancer patients and, in turn, would bolster national guidelines for genetic testing, and reduce healthcare costs. However, we cannot rule out that testing breast cancer patients with a low risk of GPVs would further increase the detection rate [57] but should be undertaken considering local healthcare resources and patient desires.

However, there are some limitations in this study. As our study included few carriers of GPVs in CPGs other than *BRCA1/2*, their endophenotypes were not well-represented. Although this study employed a multi-center design, only Chinese female patients with breast cancer were investigated. Extending the usage of this model in other ethnicities requires further tuning via training the model with ethnicity-specific dataset, following by validating in larger cohorts in the corresponding population.

## Conclusions

Breast cancer patients with GPVs in different CPGs exhibit distinct endophenotypes. Based on these distinct features, we developed and validated a phenotype-driven risk prediction model using a deep learning algorithm to identify GPV carriers among unselected breast cancer patients in a multi-center cohort. The DrABC model better predicted the risk of carrying GPVs in *BRCA1/2* or other CPGs in the Chinese population compared to previous risk prediction models which were trained in other populations. This robust germline defect risk stratification tool can be utilized to triage patients at higher risk for genetic testing.

## Supplementary Information

**Additional file 1.** Supplementary Methods.

**Additional file 2: Figure S1.** Summary of Variants in Cancer Predisposition Genes.

**Additional file 3: Table S1.** Summary of Pathogenic Variants and Variants of Uncertain Significance. 

**Additional file 4: Figure S2.** Association of Germline Variants with Age at Diagnosis (A), Family History (B), Histological Grade (C), and Molecular Subtypes (D).

**Additional file 5: Figure S3.** Phenotype-genotype correlation and data interpretation.

**Additional file 6: Figure S4.** Association of Germline Variants with Clinical Characteristics.

**Additional file 7: Figure S5.** The Multi-center Validation of the DrABC Model.

**Additional file 8:** **Table S2.** The Neurons in the Input Layer of the DrABC Model.

**Additional file 9: Figure S6.** Developing the DrABC Model through the Hierarchical Neural Network.

**Additional file 10: Figure S7.** The Performance of the DrABC Model and Other Machine Learning Models Using an Inner Five-fold Cross-validation Strategy.

**Additional file 11:** **Table S3.** The Performance of DrABC versus Previous Models in Multi-Center Validation Cohort. 

**Additional file 12:** **Table S4.** The Prediction Accuracy of the DrABC Model in Discovery and Validation Cohorts.

**Additional file 13: Figure S8.** The Distribution of the Predicted Probabilities in Non-carriers and CPGs-carriers by the DrABC Model.

**Additional file 14: Figure S9.** Performance of Risk Prediction Models for Breast Cancer Patients with germline Pathogenic Variants in Cancer Predisposition Genes other than *BRCA1/2*.

**Additional file 15: Figure S10.** The Contribution of Family Cancer History and Pathological Features to the DrABC Model.

**Additional file 16: Table S5.** The Algorithms and Variables Incorporated in the Risk Prediction Models.

**Additional file 17: Figure S11.** The Performance of Reconstructed Previous Models Which Were Trained in the Discovery Cohort and Tested in the Validation Cohort.

**Additional file 18:** **Table S6.** The Performance of Reconstructed Previous Models Which Were Trained in the Discovery Cohort and Tested in the Validation Cohort.

**Additional file 19: Figure S12.** Online Website for the DrABC Model.

**Additional file 20.** A user guide for the DrABC model.

**Additional file 21: Figure S13.** The Suggested Pipeline of Genetic Testing for Women with Breast Cancer.

**Additional file 22.** Pathogenic variants and variants of uncertain significance in this study.

## Data Availability

The supplement data that support the findings of this study are openly available in the supplementary materials. Patients provided informed consent to participate and to have variant information published; however, the consent obtained did not include consent to publish or share raw sequencing data. The anonymous genetic test reports involving this study are available upon request. We have deposited all the pathogenic/likely pathogenic variants and variants of uncertain significance in this study in the Additional file 22 and all the genetic data involving this study on Genome Variation Map [58] which are publicly accessible at https://ngdc.cncb.ac.cn/gvm/getProjectDetail?project = GVM000301 [59]. Scripts used to generate the findings in this study have been deposited on https://github.com/zhq921/DrABC [60].

## Abbreviations

GPV: Germline pathogenic variant
CPG: Cancer predisposition gene
DrABC: DNA-repair associated breast cancer
AUC: Area under the curve
NCCN: National Comprehensive Cancer Network
CHCAMS: Cancer Hospital of Chinese Academy of Medical Sciences
STROBE: Strengthening the Reporting of Observational Studies in Epidemiology
HR: Hormone receptor
ER: Estrogen receptor
PR: Progesterone receptor
AR: Androgen receptor
VUS: Variants of uncertain significance
HRR: Homologous recombinational repair
ROC: Receiver operating characteristic

## References

[1] Yap YS, Lu YS, Tamura K, Lee JE, Ko EY, Park YH (2019). Insights into breast cancer in the east vs the west: a review. JAMA Oncol.

[2] Kurian AW, Ward KC, Howlader N, Deapen D, Hamilton AS, Mariotto A (2019). Genetic testing and results in a population-based cohort of breast cancer patients and ovarian cancer patients. J Clin Oncol.

[3] Turner NC (2017). Signatures of DNA-repair deficiencies in breast cancer. N Engl J Med.

[4] Niravath P, Cakar B, Ellis M (2017). The role of genetic testing in the selection of therapy for breast cancer: a review. JAMA Oncol.

[5] Tutuncuoglu B, Krogan NJ (2019). Mapping genetic interactions in cancer: a road to rational combination therapies. Genome Med.

[6] Drohan B, Roche CA, Cusack JC, Hughes KS (2012). Hereditary breast and ovarian cancer and other hereditary syndromes: using technology to identify carriers. Ann Surg Oncol.

[7] Beitsch PD, Whitworth PW, Hughes K, Patel R, Rosen B, Compagnoni G (2019). Underdiagnosis of hereditary breast cancer: are genetic testing guidelines a tool or an obstacle?. J Clin Oncol.

[8] Weitzel JN, Lagos VI, Cullinane CA, Gambol PJ, Culver JO, Blazer KR (2007). Limited family structure and *BRCA* gene mutation status in single cases of breast cancer. JAMA..

[9] Mavaddat N, Rebbeck TR, Lakhani SR, Easton DF, Antoniou AC (2010). Incorporating tumour pathology information into breast cancer risk prediction algorithms. Breast Cancer Res.

[10] Manahan ER, Kuerer HM, Sebastian M, Hughes KS, Boughey JC, Euhus DM (2019). Consensus guidelines on genetic testing for hereditary breast cancer from the American Society of Breast Surgeons. Ann Surg Oncol.

[11] Yang S, Axilbund JE, O'Leary E, Michalski ST, Evans R, Lincoln SE (2018). Underdiagnosis of hereditary breast and ovarian cancer in medicare patients: genetic testing criteria miss the mark. Ann Surg Oncol.

[12] Daly MB, Pilarski R, Yurgelun MB, Berry MP, Buys SS, Dickson P (2020). NCCN guidelines insights: genetic/familial high-risk assessment: breast, ovarian, and pancreatic, version 1.2020. J Natl Compr Cancer Netw.

[13] Yadav S, Hu C, Hart SN, Boddicker N, Polley EC, Na J (2020). Evaluation of germline genetic testing criteria in a hospital-based series of women with breast cancer. J Clin Oncol.

[14] Milliron KJ, Griggs JJ (2019). Advances in genetic testing in patients with breast cancer, high-quality decision making, and responsible resource allocation. J Clin Oncol.

[15] Foulkes WD, Knoppers BM, Turnbull C (2016). Population genetic testing for cancer susceptibility: founder mutations to genomes. Nat Rev Clin Oncol.

[16] Bernstein-Molho R, Singer A, Laitman Y, Netzer I, Zalmanoviz S, Friedman E (2019). Multigene panel testing in unselected Israeli breast cancer cases: mutational spectrum and use of *BRCA1/2* mutation prediction algorithms. Breast Cancer Res Treat.

[17] Dias R, Torkamani A (2019). Artificial intelligence in clinical and genomic diagnostics. Genome Med.

[18] Wang X, Zou C, Zhang Y, Li X, Wang C, Ke F (2021). Prediction of *BRCA* gene mutation in breast cancer based on deep learning and histopathology images. Front Genet.

[19] Nero C, Ciccarone F, Boldrini L, Lenkowicz J, Paris I, Capoluongo ED (2020). Germline *BRCA1-2* status prediction through ovarian ultrasound images radiogenomics: a hypothesis generating study (PROBE study). Sci Rep.

[20] Mizukami K, Iwasaki Y, Kawakami E, Hirata M, Kamatani Y, Matsuda K (2020). Genetic characterization of pancreatic cancer patients and prediction of carrier status of germline pathogenic variants in cancer-predisposing genes. EBioMedicine..

[21] von Elm E, Altman DG, Egger M, Pocock SJ, Gotzsche PC, Vandenbroucke JP (2007). The strengthening the reporting of observational studies in epidemiology (STROBE) statement: guidelines for reporting observational studies. Ann Intern Med.

[22] Curigliano G, Burstein HJ, Winer EP, Gnant M, Dubsky P, Loibl S (2017). De-escalating and escalating treatments for early-stage breast cancer: the St. Gallen international expert consensus conference on the primary therapy of early breast cancer 2017. Ann Oncol.

[23] American Joint Committee on Cancer (AJCC) (2017). AJCC cancer staging manual.

[24] Wang K, Zhao S, Liu B, Zhang Q, Li Y, Liu J (2018). Perturbations of BMP/TGF-beta and VEGF/VEGFR signalling pathways in non-syndromic sporadic brain arteriovenous malformations (BAVM). J Med Genet.

[25] Zhao S, Zhang Y, Chen W, Li W, Wang S, Wang L (2021). Diagnostic yield and clinical impact of exome sequencing in early-onset scoliosis (EOS). J Med Genet.

[26] Richards S, Aziz N, Bale S, Bick D, Das S, Gastier-Foster J (2015). Standards and guidelines for the interpretation of sequence variants: a joint consensus recommendation of the American College of Medical Genetics and Genomics and the Association for Molecular Pathology. Genet Med.

[27] Li Q, Wang K (2017). InterVar: clinical interpretation of genetic variants by the 2015 ACMG-AMP guidelines. Am J Hum Genet.

[28] Danos AM, Krysiak K, Barnell EK, Coffman AC, McMichael JF, Kiwala S (2019). Standard operating procedure for curation and clinical interpretation of variants in cancer. Genome Med.

[29] Eccles DM, Mitchell G, Monteiro AN, Schmutzler R, Couch FJ, Spurdle AB (2015). *BRCA1* and *BRCA2* genetic testing-pitfalls and recommendations for managing variants of uncertain clinical significance. Ann Oncol.

[30] Spurdle AB, Healey S, Devereau A, Hogervorst FB, Monteiro AN, Nathanson KL (2012). ENIGMA--evidence-based network for the interpretation of germline mutant alleles: an international initiative to evaluate risk and clinical significance associated with sequence variation in *BRCA1* and *BRCA2* genes. Hum Mutat.

[31] Klambauer G, Unterthiner T, Mayr A, Hochreiter S (2017). Self-normalizing neural networks. Proceedings of the 31st international conference on neural information processing systems.

[32] Alvarez S, Diaz-Uriarte R, Osorio A, Barroso A, Melchor L, Paz MF (2005). A predictor based on the somatic genomic changes of the *BRCA1/BRCA2* breast cancer tumors identifies the non-*BRCA1/BRCA2* tumors with *BRCA1* promoter hypermethylation. Clin Cancer Res.

[33] Breiman L (2001). Random forests. Mach Learn.

[34] LeDell E, Poirier S (2020). H2o automl: scalable automatic machine learning. Proceedings of the AutoML workshop at ICML.

[35] Mazzola E, Blackford A, Parmigiani G, Biswas S (2015). Recent enhancements to the genetic risk prediction model BRCAPRO. Cancer Informat.

[36] Bonadona V, Sinilnikova OM, Lenoir GM, Lasset C (2002). Pretest prediction of *BRCA1* or *BRCA2* mutation by risk counselors and the computer model BRCAPRO. J Natl Cancer Inst.

[37] Frank TS, Deffenbaugh AM, Reid JE, Hulick M, Ward BE, Lingenfelter B (2002). Clinical characteristics of individuals with germline mutations in *BRCA1* and *BRCA2*: analysis of 10,000 individuals. J Clin Oncol.

[38] Lindor NM, Johnson KJ, Harvey H, Shane Pankratz V, Domchek SM, Hunt K (2010). Predicting *BRCA1* and *BRCA2* gene mutation carriers: comparison of PENN II model to previous study. Familial Cancer.

[39] Lee A, Mavaddat N, Wilcox AN, Cunningham AP, Carver T, Hartley S (2019). BOADICEA: a comprehensive breast cancer risk prediction model incorporating genetic and nongenetic risk factors. Genet Med.

[40] DeLong ER, DeLong DM, Clarke-Pearson DL (1988). Comparing the areas under two or more correlated receiver operating characteristic curves: a nonparametric approach. Biometrics..

[41] Sun X, Xu W (2014). Fast implementation of DeLong’s algorithm for comparing the areas under correlated receiver operating characteristic curves. IEEE Signal Proc Lett.

[42] Reiser B (2000). Measuring the effectiveness of diagnostic markers in the presence of measurement error through the use of ROC curves. Stat Med.

[43] Paluch-Shimon S, Pagani O, Partridge AH, Abulkhair O, Cardoso MJ, Dent RA (2017). ESO-ESMO 3rd international consensus guidelines for breast cancer in young women (BCY3). Breast..

[44] Zhao W, Wiese C, Kwon Y, Hromas R, Sung P (2019). The *BRCA* tumor suppressor network in chromosome damage repair by homologous recombination. Annu Rev Biochem.

[45] Couch FJ, Shimelis H, Hu C, Hart SN, Polley EC, Na J (2017). Associations between cancer predisposition testing panel genes and breast cancer. JAMA Oncol.

[46] Cheon JY, Mozersky J, Cook-Deegan R (2014). Variants of uncertain significance in *BRCA*: a harbinger of ethical and policy issues to come?. Genome Med.

[47] Lee AJ, Cunningham AP, Kuchenbaecker KB, Mavaddat N, Easton DF, Antoniou AC (2014). BOADICEA breast cancer risk prediction model: updates to cancer incidences, tumour pathology and web interface. Br J Cancer.

[48] Kurian AW, Ward KC, Abrahamse P, Hamilton AS, Deapen D, Morrow M, et al. Association of germline genetic testing results with locoregional and systemic therapy in patients with breast cancer. JAMA Oncol. 2020;6(4):e196400. 10.1001/jamaoncol.2019.6400 PMC7042883 32027353

[49] Eoh KJ, Park JS, Park HS, Lee ST, Han J, Lee JY (2017). *BRCA1* and *BRCA2* mutation predictions using the BRCAPRO and myriad models in Korean ovarian cancer patients. Gynecol Oncol.

[50] James PA, Doherty R, Harris M, Mukesh BN, Milner A, Young MA (2006). Optimal selection of individuals for *BRCA* mutation testing: a comparison of available methods. J Clin Oncol.

[51] Barcenas CH, Hosain GM, Arun B, Zong J, Zhou X, Chen J (2006). Assessing *BRCA* carrier probabilities in extended families. J Clin Oncol.

[52] Lupski JR, Belmont JW, Boerwinkle E, Gibbs RA (2011). Clan genomics and the complex architecture of human disease. Cell..

[53] Hung FH, Wang YA, Jian JW, Peng HP, Hsieh LL, Hung CF (2019). Evaluating *BRCA* mutation risk predictive models in a Chinese cohort in Taiwan. Sci Rep.

[54] Youlden DR, Cramb SM, Yip CH, Baade PD (2014). Incidence and mortality of female breast cancer in the Asia-Pacific region. Cancer Biol Med.

[55] Zhang J, Pei R, Pang Z, Ouyang T, Li J, Wang T (2012). Prevalence and characterization of *BRCA1* and *BRCA2* germline mutations in Chinese women with familial breast cancer. Breast Cancer Res Treat.

[56] Kim H, Choi DH (2013). Distribution of *BRCA1* and *BRCA2* mutations in Asian patients with breast cancer. J Breast Cancer.

[57] Kurian AW, Bernhisel R, Larson K, Caswell-Jin JL, Shadyab AH, Ochs-Balcom H (2020). Prevalence of pathogenic variants in cancer susceptibility genes among women with postmenopausal breast cancer. JAMA..

[58] Song S, Tian D, Li C, Tang B, Dong L, Xiao J (2018). Genome variation map: a data repository of genome variations in BIG data center. Nucleic Acids Res.

[59] Zhao H. GVM000301. Genome Variation Map. URL: https://ngdc.cncb.ac.cn/search/?dbId=gvm&q=GVM000301. Accessed 11 Jan 2022.

[60] Liu J, Zhao H. The DNA-repair pathway Associated Breast Cancer (DrABC) calculator scripts. Github. URL: https://github.com/zhq921/DrABC. Accessed 20 Dec 2021.

