# Supplementary material for: DrABC: deep learning accurately predicts germline pathogenic mutation status in breast cancer patients based on phenotype data
Source: Genome Med. 2022 Feb 25;14:21. doi: 10.1186/s13073-022-01027-9 (PMC8876403; doi:10.1186/s13073-022-01027-9)
Supplement: Supplementary file 1 — Additional file 1. Supplementary Methods. [file 13073_2022_1027_MOESM1_ESM.pdf]

Supplementary Methods

Liu et al. DrABC: Deep Learning Accurately Predicts Germline Pathogenic Mutation Status in Breast Cancer Patients Based on Phenotype Data

Supplementary method 1. Patient Recruitment 2

Supplementary method 2. Patient Phenotyping 2

Supplementary method 3. Sample Collection and DNA Extractions 3

Supplementary method 4. Panel-Based Sequencing Assay 3

Supplementary method 5. Variant-Calling and Annotation 4

Supplementary method 6. Single Nucleotide Variants (SNVs) /Indels Interpretation 5

Supplementary method 7. Missing Data Imputation in the DrABC Model 6

Supplementary method 8. The Ensemble Learning Design of the DrABC Model 6

Supplementary method 9. Calculation of GPV Carrier Probabilities Using Previous Models.. 7

References in the Supplement: 9

This supplementary material has been provided by the authors to give readers additional information about their work.

Supplementary method 1. Patient Recruitment

This multicenter cohort study recruited consecutive female patients with breast cancer from October 1, 2017, to August 31, 2019, at the Cancer Hospital of Chinese Academy of Medical Sciences and Peking Union Medical College (the main patient source) and other six hospitals, including 1) Huanxing Cancer Hospital, 2) Guiyang Maternal and Child Healthcare Hospital in Guiyang, 3) the Affiliated Cancer Hospital of Zhengzhou University, 4) the Affiliated Yantai Yuhuangding Hospital of Qingdao University, 5) the Fourth Hospital of Hebei Medical University, 6) Beijing Tiantan Hospital all in China. The study was reviewed and approved by the ethics committee of each participating hospital. Written informed consent was obtained from each participant.

Supplementary method 2. Patient Phenotyping

We collected phenotypic data including the age at diagnosis, family history, cancer history, pathology features, molecular subtype, and clinical stage. The cancer history and family history for each case were taken by trained clinicians or genetic counselors based on information provided by the patients and/or their family members. The positive hormone receptor (HR), including estrogen receptor (ER) and progesterone receptor (PR), was defined as more than 1% of tumor cells stain positive for estrogen receptor or progesterone receptor proteins. Similar to HR, androgen receptor (AR)-positive was defined as more than 1% of tumor cells stain positive. The ERBB2/HER2 positive was defined as tumor cells stain strongly (3+) for ERBB2 protein or *ERBB2* gene is amplified in tumor cells. Triple-negative was defined as a tumor that does not meet any pathologic criteria for positivity of estrogen receptor, progesterone receptor, or ERBB2/HER2. (1) Clinical grouping of molecular subtypes was defined by the status of hormone receptor and HER2 according to the St. Gallen 2017 criteria(2). The staging was determined by the status of the

primary tumor (T), lymph node (N), and metastasis (M) according to the eighth edition of classification for BC of the American Joint Commission of Cancer (AJCC). (3)

Supplementary method 3. Sample Collection and DNA Extractions

Saliva samples or approximately 2 mL of peripheral blood were collected from all participants. Genomic DNA was extracted from the whole blood and saliva samples with the QIAamp DNA Mini Kit (QIAGEN, Germany) and TGuide Genomic DNA OneStep Kit using a TGuide automated Nucleic Acid Preparation Instrument (TIANGEN BIOTECH, Beijing, China), respectively. The quality and concentration of the DNA samples were determined with a dsDNA HS Assay Kit using a Qubit 2.0 Fluorometer (Thermo Fisher Scientific, CA, USA). Genomic DNA was stored at -20 °C.

Supplementary method 4. Panel-Based Sequencing Assay

Genomic DNA in amount of 200 ng was fragmented using a Covaris M220 sonicator (Covaris Inc., Woburn, MA, USA). After the fragmentation process, end-repair, A-tailing, adapter ligation, PCR reactions, and target enrichment were performed, following the manufacturer's recommended protocols from the Agilent SureSelect-XT Low Input Target Enrichment kit (Agilent Technologies, USA). Molecular barcoded DNA libraries were hybridized with a commercial ClearSeq Inherited Disease multigene panel, which covered total exons and intron boundaries within at least ±20 bases of cancer predisposition genes, including *ATM, BARD1, BRIP1, BRCA1, BRCA2, CDH1, PALB2, RAD5IC, RAD51D, CHEK2, NBN, TP53, PTEN, STKI1, APC, MUTYH, MLH1, MSH2, MSH6, PMS2, SMAD4, KIT, PDGFA, HOXB13, RB1, PTCH1, CDK4, CDKN2A, PALLD, WRN, MEN1, RECQL, RET, SDHA, SDHB, SDHC, SDHD, SDHAF2, GNAS, MAX, VHL, MET, FH, FLCN, TSC1, TSC2, PRKAR1A, SMARCA4, SMARCB1,* and *BRAF* (Agilent Technologies). Final libraries were quantified using a Qubit High Sensitivity kit (Thermo

Fisher Scientific), and the quality of the library was assessed using a Bioanalyzer High Sensitivity DNA chip (Agilent Technologies, USA). DNA libraries were sequenced using an Illumina HiSeq 4000 instrument (Illumina, USA).

Supplementary method 5. Variant-Calling and Annotation

The variant-calling and annotation steps were performed using the in-house developed PUMP (Peking Union Medical College hospital Pipeline)(4). Valid reads were aligned to the reference human genome GRCh37/hg19 using Burrows-Wheeler Aligner (BWA) software. Single-nucleotide variants and small insertions and/or deletions (aka indels) were called using the HaplotypeCaller of the Genome Analysis Toolkit (GATK), version 3.4.0. The SAMtools suite (http://samtools.sourceforge.net) was also used to call single-nucleotide variants and short insertions/deletions (<30 base pairs) as supplementary. Computational prediction tools (GERP++(5), Combined Annotation Dependent Depletion (CADD)(6), SIFT(7), Polyphen-2(8), and VariantTaster(9)) were used to predict the conservation and pathogenicity of candidate variants. All variants were compared against publicly available databases such as the 1000 Genomes Project (http://www.internationalgenome.org/), the Exome variant server, NHLBI GO Exome Sequencing Project (ESP) (http://evs.gs.washington.edu/EVS/), and the Exome Aggregation Consortium (ExAC; http://exac.broadinstitute.org/). We used ANNOVAR(10) to annotate the frequency of each allele in the ExAC database, location of the variant within genes (including exonic, splice site, nearby intronic, and intragenic), and predicted consequence for all variants. Candidate variants were then restricted to rare variants (ExAC_MAF<=0.001) that altered the coding sequence (missense, nonsense, splice-site, frameshift, and in-frame indels). The resulting variants were further annotated with current information available from ClinVar (http://www.ncbi.nlm.nih.gov/clinvar/)(11) and the Online Mendelian Inheritance in Man

(OMIM)(12) databases using custom perl scripts. All variants were double-checked using the AlamutVisual software, which was an integrated platform for the clinical interpretation of genomic variants. Sequencing quality of selected variants was checked in the Integrative Genomics Viewer V2.3 (IGV) (13) to rule out false positives, and likely casual candidates were confirmed by Sanger sequencing. Computational CNV calling was based on next-generation sequencing data independently using the software XHMM(14). The coverage information was computed from BAM files using GATK. Then, extreme targets or samples were filtered out. Highly variable targets were also filtered out. After quality control, read depths for each sample were analyzed and a z score was calculated. CNVs were called according to the z-score.(14)

Supplementary method 6. Single Nucleotide Variants (SNVs) /Indels Interpretation

The protocol for interpretation of SNVs and indels was adapted from the American College of Medical Genetics and Genomics and the Association for Molecular Pathology (ACMG/AMP) guidelines (15), with the assist of InterVar (http://wintervar.wglab.org/) (16). Variants annotated by the PUMP pipeline (4) were first filtered against a population frequency of 0.1% based on 1000 Genomes (October 2013) and the genome Aggregation Database (gnomAD, http://gnomad.broadinstitute.org/). Pathogenicity of retained variants was evaluated according to variant type, reported evidence, function prediction software, and presence of informative *in trans* allele. Variants predicted to cause protein truncation (including stop-gain, frameshift, canonical splice acceptor/donor variants) meeting PVS1 criteria (17), were classified as pathogenic variants/likely pathogenic. However, we excluded the truncating variants in the last 55bp of the penultimate exon or the last exon, which might avoid nonsense-mediated mRNA decay. The variants in *BRCA1/2* were further analyzed according to the Evidence-based Network for the Interpretation of Germline Mutant Alleles (ENIGMA) consortium (https://enigmaconsortium.org/)

(18, 19). For those variants without available expert panel results, the results of annotation were compared with classifications in ClinVar (https://www.ncbi.nlm.nih.gov/clinvar/) to identify additional information and to determine the final classification of each variant, collapsed from a 5-tier to 3-tier classification system of pathogenic/likely pathogenic, benign/likely benign, and uncertain significance. Variants classified as pathogenic or likely pathogenic were considered pathogenic in this study, those classified as benign or likely benign were considered benign. All intronic and synonymous variants were defined as benign. For patients who carried more than one variant, the variant type was classified as the most damaging one.

Supplementary method 7. Missing Data Imputation in the DrABC Model

To derive a more robust and accurate model, patients without key clinical information (including the age at diagnosis, personal cancer history, and family cancer history; Table S2) were excluded from the construction of the DrABC model. However, the missing data of the pathological features are tolerant by handling the missing data to prevent data loss. As numerical continuous variables and categorical variables were both present in our scenario, the missing data is imputed using proximity from the random forest algorithm (20). For continuous predictors, the imputed value is the weighted average of the non-missing observations, where the weights are the proximities. For categorical predictors, the imputed value is the category with the largest average proximity. This process is iterated five times. The imputation of missing data was performed using R statistical software, version 3.5.1.

Supplementary method 8. The Ensemble Learning Design of the DrABC Model

To obtain better predictive performance, we used ensemble learning methods that combine 101 deep learning algorithms into a single predictive model. Ensemble learning, one of the most powerful machine learning techniques, is the process of training a set of machine learning models

and combining their outputs together. Combining a diverse set of individual machine learning models can improve the stability of the overall model, leading to more accurate predictions. The basic concept behind this is that a group of “weak learners”, which are even just slightly better than a random guess, may come together to build a “strong learner”. Ensemble learning methods often place first in circumstances requiring machine learning, such as random forest(20, 21), a widely used algorithm for classification. In this study, we trained a set of deep learning models (n = 101) on different subsets of the training data, and aggregated them into a final model predicting the class labels using the majority vote, i.e., each patient was assigned with a category with the highest frequency output in all 101 deep learning models. Due to the random subset of the training data employed in each model, the number of models to develop should not be set too small to ensure that every patient gets predicted at least a few times. As a result, 101 were selected to build the ensemble learning algorithm considering the balance between predictive performance and computational performance.

Supplementary method 9. Calculation of GPV Carrier Probabilities Using Previous Models

The risk of carrying a GPV in *BRCA1/2* or CPGs was also estimated using NCCN guidelines (version 1.2020) (22, 23), BRCAPRO (version 2.1-7) (24, 25), Myriad II (26), PENN II (https://pennmodel2.pmacs.upenn.edu/penn2/) (27), and BOADICEA (v3) (28) models in the multi-center validation cohort. The inputs for the NCCN guidelines, the Myriad model, and the Penn II were a summary of personal and family cancer history. The Penn II model was conducted using a web interface (https://pennmodel2.pmacs.upenn.edu/penn2/). The Myriad model only predicted the risk of carrying a GPV in *BRCA1/2* through a mutation prevalence table downloaded from the Myriad Genetics website (https://webapps.myriad.com/brca-risk-calculator/). The BayesMendel R package version 2.1–7 of the BRCAPRO model

(https://projects.iq.harvard.edu/bayesmendel/bayesmendel-r-package/) was conducted by inputting of the cancer history and age at diagnosis of each patient and her relatives. For BOADICEA, more information was needed to generate the of carrying a GPV in CPGs, including the cancer history, age at diagnosis, and the breast cancer pathological features (ER/PR/HER2) of the proband and the cancer history and age at diagnosis of her relatives, using the BOADICEA web application v3, (https://pluto.srl.cam.ac.uk/cgi-bin/bd3/v3/bd.cgi).

References in the Supplement:

1. Waks AG, Winer EP. Breast cancer treatment: A review. JAMA. 2019;321(3):288-300.

2. Curigliano G, Burstein HJ, Winer EP, Gnant M, Dubsky P, Loibl S, et al. De-escalating and escalating treatments for early-stage breast cancer: the St. Gallen International Expert Consensus Conference on the Primary Therapy of Early Breast Cancer 2017. Ann Oncol. 2017;28(8):1700-12.

3. American Joint Committee on Cancer (AJCC). AJCC Cancer Staging Manual. 8th ed. New York: Springer; 2017.

4. Zhao S, Zhang Y, Chen W, Li W, Wang S, Wang L, et al. Diagnostic yield and clinical impact of exome sequencing in early-onset scoliosis (EOS). J Med Genet. 2021;58(1):41-7.

5. Davydov EV, Goode DL, Sirota M, Cooper GM, Sidow A, Batzoglou S. Identifying a high fraction of the human genome to be under selective constraint using GERP++. PLoS Comput Biol. 2010;6(12):e1001025.

6. Kircher M, Witten DM, Jain P, O'Roak BJ, Cooper GM, Shendure J. A general framework for estimating the relative pathogenicity of human genetic variants. Nat Genet. 2014;46(3):310-5.

7. Vaser R, Adusumalli S, Leng SN, Sikic M, Ng PC. SIFT missense predictions for genomes. Nat Protoc. 2016;11(1):1-9.

8. Adzhubei IA, Schmidt S, Peshkin L, Ramensky VE, Gerasimova A, Bork P, et al. A method and server for predicting damaging missense mutations. Nat Methods. 2010;7(4):248-9.

9. Schwarz JM, Cooper DN, Schuelke M, Seelow D. MutationTaster2: mutation prediction for the deep-sequencing age. Nat Methods. 2014;11(4):361-2.

10. Wang K, Li M, Hakonarson H. ANNOVAR: functional annotation of genetic variants from high-throughput sequencing data. Nucleic Acids Res. 2010;38(16):e164.

11. Landrum MJ, Lee JM, Benson M, Brown GR, Chao C, Chitipiralla S, et al. ClinVar: improving access to variant interpretations and supporting evidence. Nucleic Acids Res. 2018;46(D1):D1062-D7.

12. OMIM®. Online Mendelian Inheritance in Man Baltimore, MD: McKusick-Nathans Institute of Genetic Medicine, Johns Hopkins University; 2018 [Available from: https://omim.org/.

13. Thorvaldsdottir H, Robinson JT, Mesirov JP. Integrative Genomics Viewer (IGV): high-performance genomics data visualization and exploration. Brief Bioinform. 2013;14(2):178-92.

14. Fromer M, Moran JL, Chambert K, Banks E, Bergen SE, Ruderfer DM, et al. Discovery and statistical genotyping of copy-number variation from whole-exome sequencing depth. Am J Hum Genet. 2012;91(4):597-607.

15. Richards S, Aziz N, Bale S, Bick D, Das S, Gastier-Foster J, et al. Standards and guidelines for the interpretation of sequence variants: a joint consensus recommendation of the American College of Medical Genetics and Genomics and the Association for Molecular Pathology. Genet Med. 2015;17(5):405-24.

16. Li Q, Wang K. InterVar: Clinical Interpretation of Genetic Variants by the 2015 ACMG-AMP Guidelines. Am J Hum Genet. 2017;100(2):267-80.

17. Abou Tayoun AN, Pesaran T, DiStefano MT, Oza A, Rehm HL, Biesecker LG, et al. Recommendations for interpreting the loss of function PVS1 ACMG/AMP variant criterion. Hum Mutat. 2018;39(11):1517-24.

18. Eccles DM, Mitchell G, Monteiro AN, Schmutzler R, Couch FJ, Spurdle AB, et al. *BRCA1* and *BRCA2* genetic testing-pitfalls and recommendations for managing variants of uncertain clinical significance. Ann Oncol. 2015;26(10):2057-65.

19. Spurdle AB, Healey S, Devereau A, Hogervorst FB, Monteiro AN, Nathanson KL, et al. ENIGMA--evidence-based network for the interpretation of germline mutant alleles: an international initiative to evaluate risk and clinical significance associated with sequence variation in *BRCA1* and *BRCA2* genes. Hum Mutat. 2012;33(1):2-7.

20. Breiman L. Random Forests. Machine Learning. 2001;45(1):5-32.

21. Alvarez S, Diaz-Uriarte R, Osorio A, Barroso A, Melchor L, Paz MF, et al. A predictor based on the somatic genomic changes of the *BRCA1/BRCA2* breast cancer tumors identifies the non-*BRCA1/BRCA2* tumors with *BRCA1* promoter hypermethylation. Clin Cancer Res. 2005;11(3):1146-53.

22. NCCN Clinical Practice Guidelines in Oncology. Genetic/familial high-risk assessment: Breast, ovarian, and pancreatic. Version 1. https://www.nccn.org/professionals/physician_gls/pdf/genetics_bop.pdf. 2020.

23. Daly MB, Pilarski R, Yurgelun MB, Berry MP, Buys SS, Dickson P, et al. NCCN guidelines insights: Genetic/familial high-risk assessment: breast, ovarian, and pancreatic, Version 1.2020. J Natl Compr Canc Netw. 2020;18(4):380-91.

24. Mazzola E, Blackford A, Parmigiani G, Biswas S. Recent enhancements to the genetic risk prediction model BRCAPRO. Cancer Inform. 2015;14(Suppl 2):147-57.

25. Bonadona V, Sinilnikova OM, Lenoir GM, Lasset C. Pretest prediction of *BRCA1* or *BRCA2* mutation by risk counselors and the computer model BRCAPRO. J Natl Cancer Inst. 2002;94(20):1582-3; author reply 3-4.

26. Frank TS, Deffenbaugh AM, Reid JE, Hulick M, Ward BE, Lingenfelter B, et al. Clinical characteristics of individuals with germline mutations in *BRCA1* and *BRCA2*: analysis of 10,000 individuals. J Clin Oncol. 2002;20(6):1480-90.

27. Lindor NM, Johnson KJ, Harvey H, Shane Pankratz V, Domchek SM, Hunt K, et al. Predicting *BRCA1* and *BRCA2* gene mutation carriers: comparison of PENN II model to previous study. Fam Cancer. 2010;9(4):495-502.

28. Lee A, Mavaddat N, Wilcox AN, Cunningham AP, Carver T, Hartley S, et al. BOADICEA: a comprehensive breast cancer risk prediction model incorporating genetic and nongenetic risk factors. Genet Med. 2019;21(8):1708-18.
