# Supplementary material for: DrABC: deep learning accurately predicts germline pathogenic mutation status in breast cancer patients based on phenotype data
Source: Genome Med. 2022 Feb 25;14:21. doi: 10.1186/s13073-022-01027-9 (PMC8876403; doi:10.1186/s13073-022-01027-9)
Supplement: Supplementary file 2 — Additional file 2: Figure S1. Summary of Variants in Cancer Predisposition Genes. [file 13073_2022_1027_MOESM2_ESM.pdf]

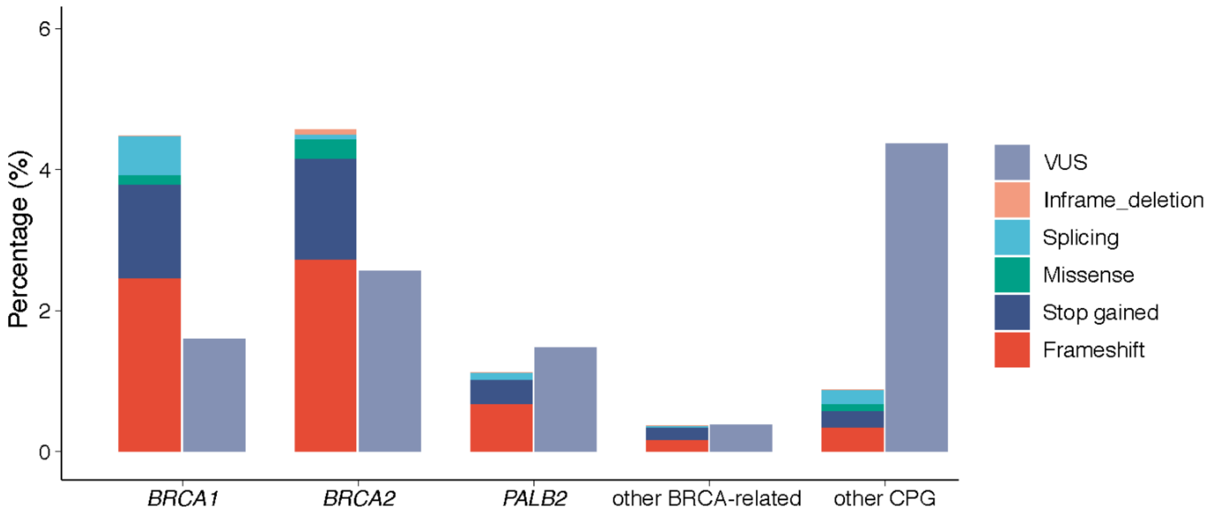

Fig. S1. Summary of Variants in Cancer Predisposition Genes.

In total, 332 (11.3%, 332/2930) patients were found to harbor germline pathogenic variants in cancer predisposition genes (CPGs). In addition, 297 variants of uncertain significance were found in 249 (8.5%) patients. As the most commonly mutated gene in Chinese BC patients, *BRCA2* was found to harbor pathogenic changes in 134 (4.6%) patients. Pathogenic variants in *BRCA1* were found in 131 (4.5%) patients. There were 33 (1.1%) patients with pathogenic variants in *PALB2*, 11 (0.4%) patients with pathogenic variants in other homologous recombinational repair (HRR)-related genes, and 26 (0.9%) with pathogenic variants in other CPGs. Frameshift variants were the most common type of pathogenic variants in *BRCA1* (2.5%), *BRCA2* (2.7%), *PALB2* (0.7%), other HRR-related genes (0.2%), and other CPGs (0.3%). There were more variants of uncertain significance in other CPGs other than the BRCA-related genes (4.4%), which might be caused by the limited public data of these genes. Additionally, the recurrent *CHEK2* c.1240T>C was found in 22 patients and *RECQL* c.2T>C was found in 12 patients.
