# Supplementary material for: DrABC: deep learning accurately predicts germline pathogenic mutation status in breast cancer patients based on phenotype data
Source: Genome Med. 2022 Feb 25;14:21. doi: 10.1186/s13073-022-01027-9 (PMC8876403; doi:10.1186/s13073-022-01027-9)
Supplement: Supplementary file 4 — Additional file 4: Figure S2. Association of Germline Variants with Age at Diagnosis (A), Family History (B), Histological Grade (C), and Molecular Subtypes (D). [file 13073_2022_1027_MOESM4_ESM.pdf]

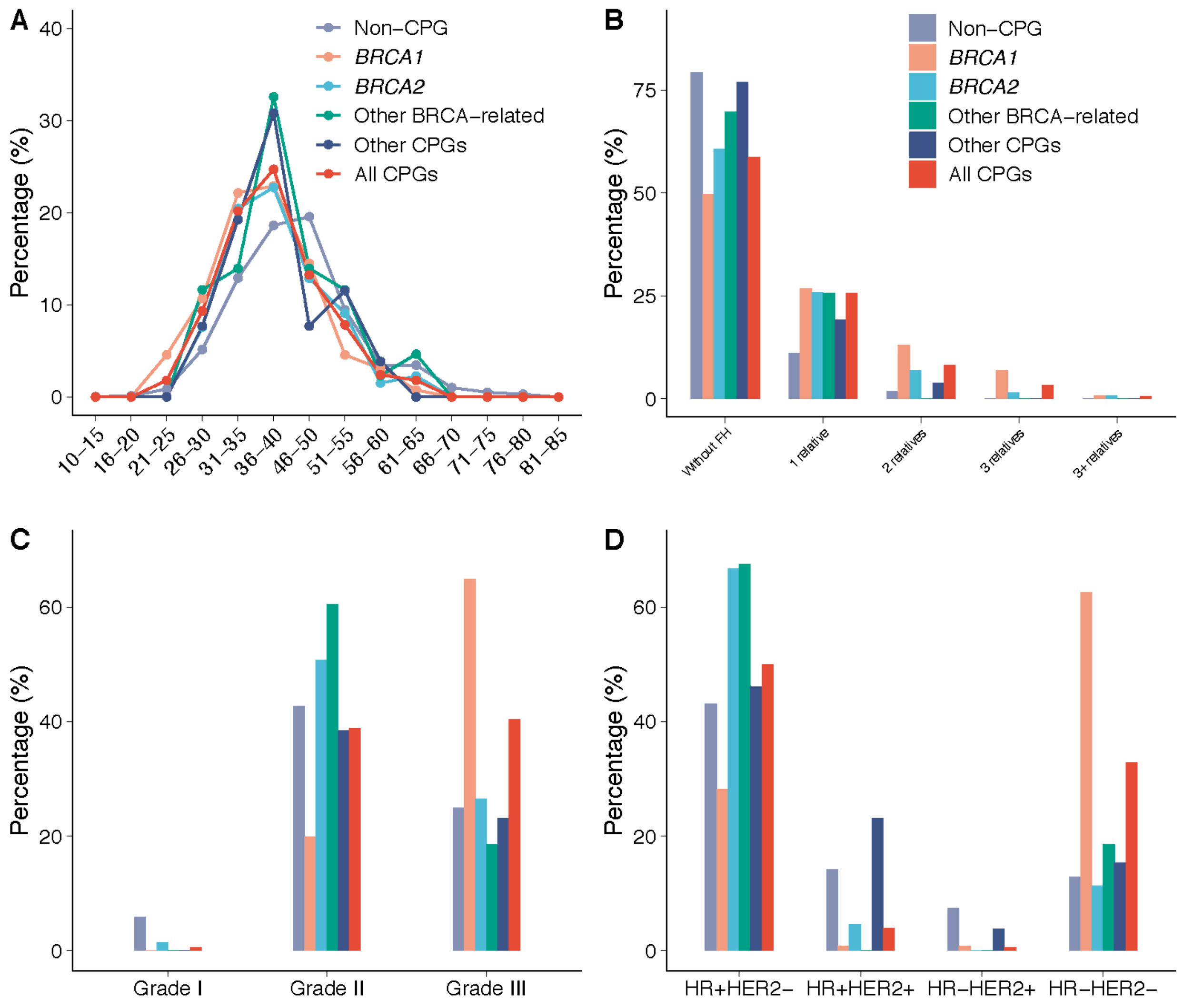

Fig. S2. Association of Germline Variants with Age at Diagnosis (A), Family History (B), Histological Grade (C), and Molecular Subtypes (D).

A) The age of diagnosis is significantly younger in patients with germline pathogenic variants (GPVs) as compared with patients without GPVs (43.43±9.08 vs. 40.15±8.29, respectively, p=5.7×10^{-10}), and even younger in patients with GPVs of *BRCA1/2*. **B)** The proportions of having one or more relatives with relevant cancers (breast cancer, ovarian cancer, pancreas cancer, and prostate cancer) were higher in patients carrying *BRCA1*, *BRCA2*, other HRR-related genes, and

other cancer predisposition genes (CPGs) than the non-carriers. A positive family history of breast cancer and all kinds of cancers were also observed in more patients with GPVs in *BRCA1/2* than in patients without GPVs (for all cancer types, 64.9% and 53.8% in *BRCA1/2* carriers and 30.9% in non-carriers, $p=1.5\times10^{-7}$). **C)** In patients with GPVs in *BRCA1*, there was less proportion of histological grade I and II than patients without GPVs (0% in grade I and 19.85% in grade II in *BRCA1* carriers vs. 5.92% and 42.78% in non-carriers, $p=6.8\times10^{-4}$ and 8.4×10^{-8}, respectively), but a higher proportion of grade III (64.89% in *BRCA1* carriers vs. 25.01% in non-carriers, $p=1.5\times10^{-20}$). Compare to the patients without GPVs, less grade I in patients with PGVs in *BRCA2* (5.92% vs. 1.52%, $p=0.03$) and more grade II in patients with PGVs in other HRR-related genes (42.78% vs. 60.47%, $p=0.03$) were identified. **D)** For the molecular subtype, more triple negative BCs were found in the *BRCA1* subgroup than the non-carriers (62.6% vs. 12.9%, $p=6.8\times10^{-37}$). However, the majorities of *BRCA2* and other HRR-related genes carriers were HR-positive and HER2-negative (66.67% and 67.44%, respectively).
