# Supplementary material for: DrABC: deep learning accurately predicts germline pathogenic mutation status in breast cancer patients based on phenotype data
Source: Genome Med. 2022 Feb 25;14:21. doi: 10.1186/s13073-022-01027-9 (PMC8876403; doi:10.1186/s13073-022-01027-9)
Supplement: Supplementary file 5 — Additional file 5: Figure S3. Phenotype-genotype correlation and data interpretation. [file 13073_2022_1027_MOESM5_ESM.pdf]

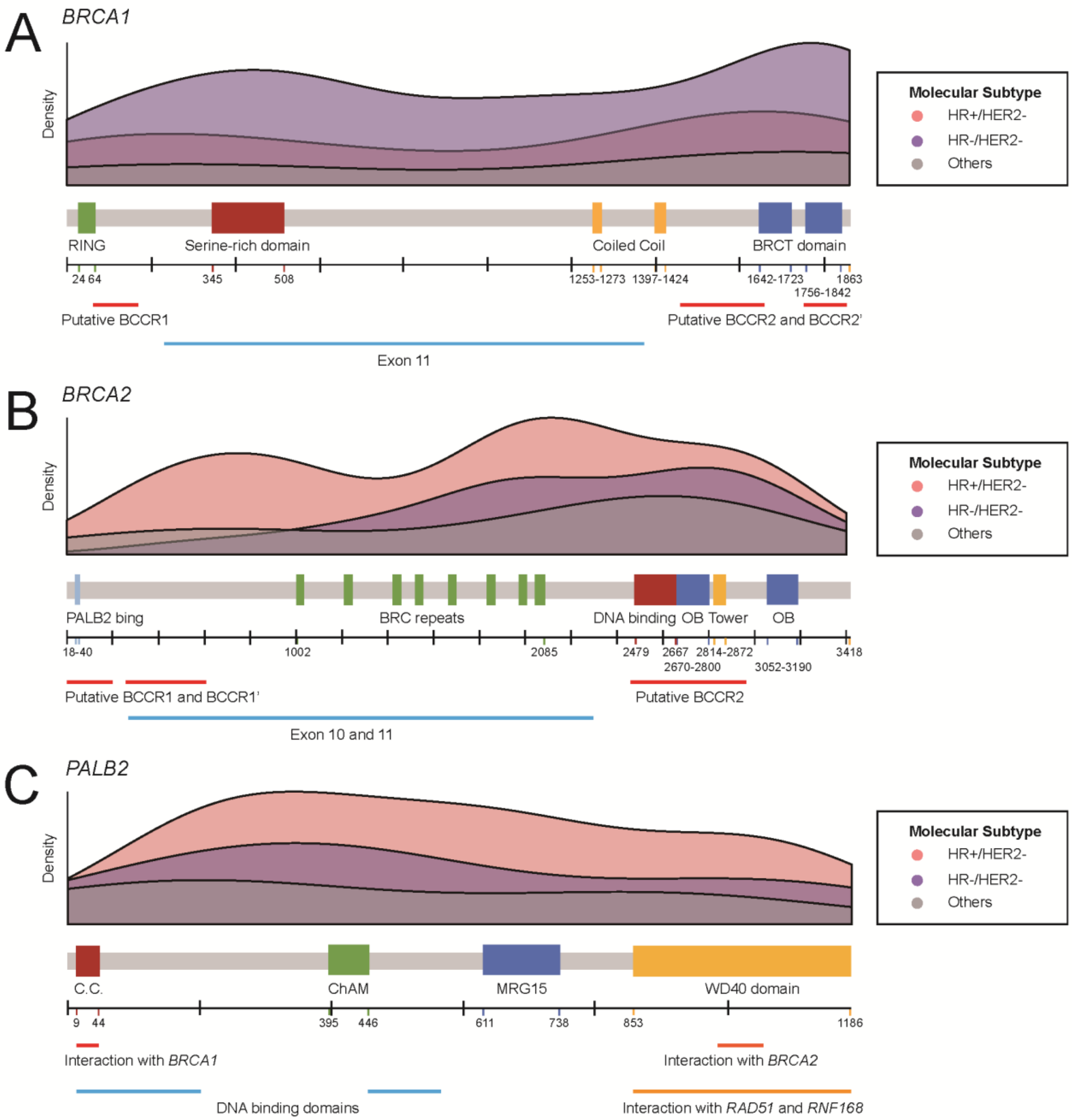

Fig. S3. Phenotype-genotype correlation and data interpretation

A) For the molecular subtype, more triple negative breast cancers were found in the *BRCA1* subgroup than the non-carriers (62.6% vs. 12.9%, $p=6.8\times10^{-37}$). **B-C)** However, the majorities of *BRCA2* **(B)** and other *PALB2* **(C)** carriers were HR-positive and HER2-negative (67.2% and 63.6%,

respectively). The pathogenic variants of the patients with triple negative breast cancers were enriched around the motifs of these genes.
