# Supplementary material for: DrABC: deep learning accurately predicts germline pathogenic mutation status in breast cancer patients based on phenotype data
Source: Genome Med. 2022 Feb 25;14:21. doi: 10.1186/s13073-022-01027-9 (PMC8876403; doi:10.1186/s13073-022-01027-9)
Supplement: Supplementary file 6 — Additional file 6: Figure S4. Association of Germline Variants with Clinical Characteristics. [file 13073_2022_1027_MOESM6_ESM.pdf]

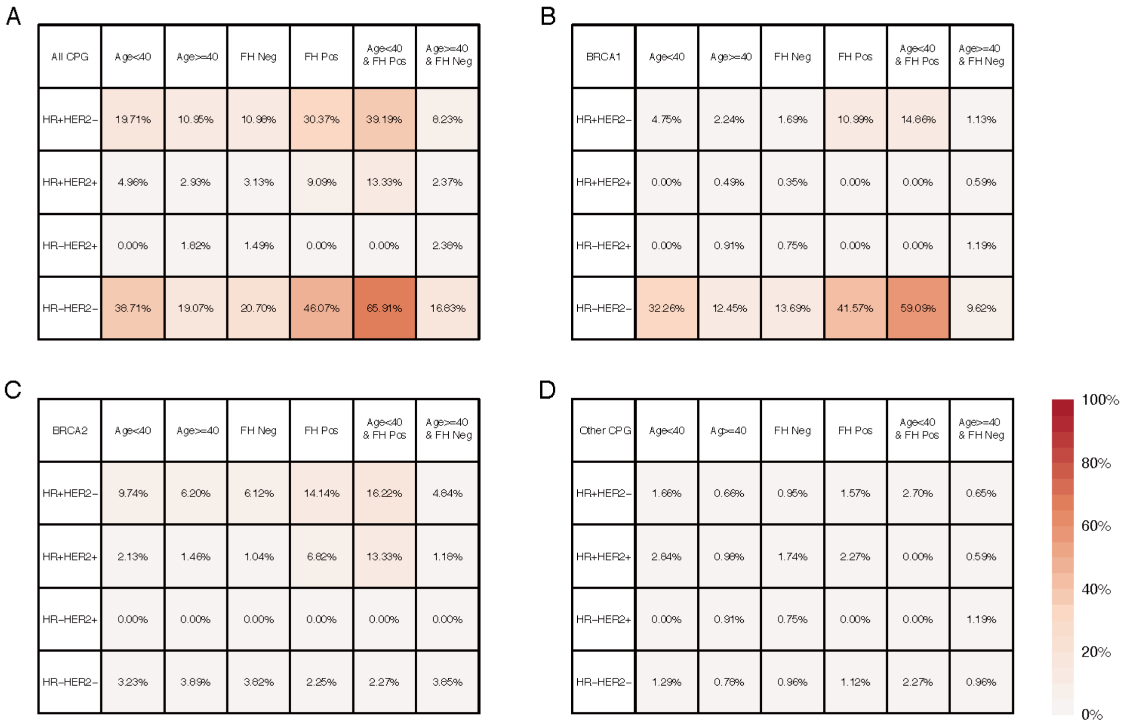


**Fig. S4. Association of Germline Variants with Clinical Characteristics**

Combing the molecular subtype with age at diagnosis and family history, patients with triple negative breast cancers diagnosed before 40 years old and positive cancer history had the highest proportion of germline pathogenic variants (GPVs) carriers (65.9%), mostly *BRCA1* carriers (59.1%). Patients with hormone receptor (HR) positive and HER2 negative breast cancer diagnosed before 40 years old and positive cancer history also had a high proportion of GPVs carriers (39.2%), including *BRCA1* carriers (14.9%) and *BRCA2* carriers (16.2%). Patients with both HR and HER2 negative breast cancer diagnosed before 40 years old and positive cancer history had a proportion of 13.3% for GPVs carriers, who were all *BRCA2* carriers.
