# Supplementary material for: DrABC: deep learning accurately predicts germline pathogenic mutation status in breast cancer patients based on phenotype data
Source: Genome Med. 2022 Feb 25;14:21. doi: 10.1186/s13073-022-01027-9 (PMC8876403; doi:10.1186/s13073-022-01027-9)
Supplement: Supplementary file 7 — Additional file 7: Figure S5. The Multi-center Validation of the DrABC Model. [file 13073_2022_1027_MOESM7_ESM.pdf]

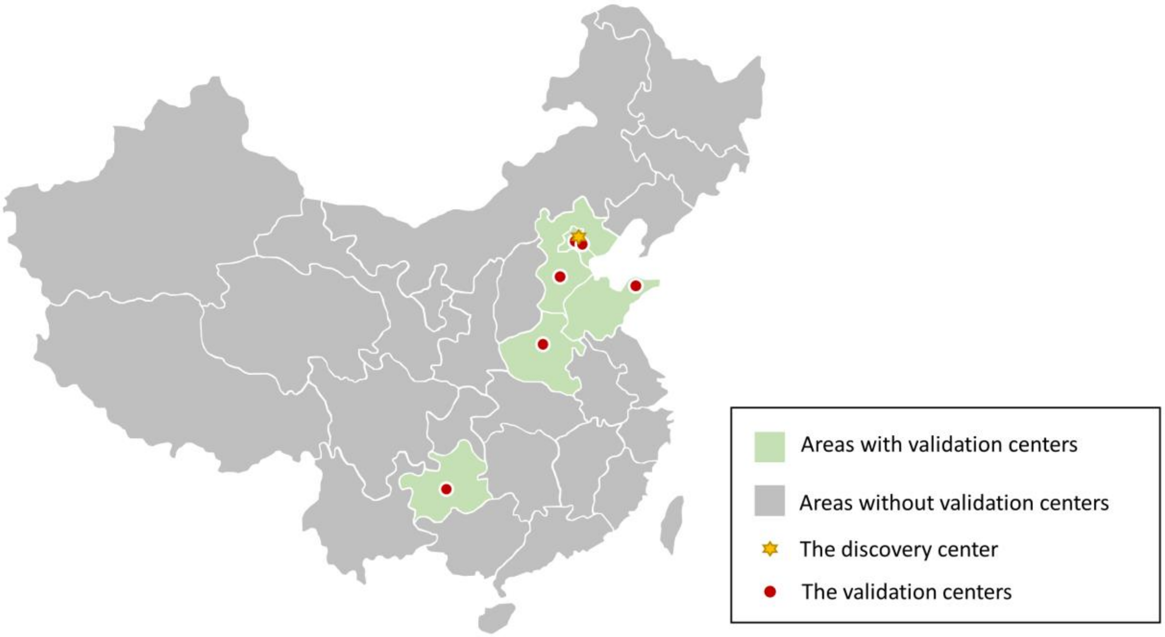

Fig. S5. The Multi-center Validation of the DrABC Model.

The discovery cohort was constituted of 1701 patients with BC from Cancer Hospital of Chinese Academy of Medical Sciences and Peking Union Medical College (CHCAMS), and 731 patients from 6 independent medical centers (red points) from 5 provinces and municipalities (green) in China. constituted the independent multi-center validation cohort.
