# Supplementary material for: DrABC: deep learning accurately predicts germline pathogenic mutation status in breast cancer patients based on phenotype data
Source: Genome Med. 2022 Feb 25;14:21. doi: 10.1186/s13073-022-01027-9 (PMC8876403; doi:10.1186/s13073-022-01027-9)
Supplement: Supplementary file 9 — Additional file 9: Figure S6. Developing the DrABC Model through the Hierarchical Neural Network. [file 13073_2022_1027_MOESM9_ESM.pdf]

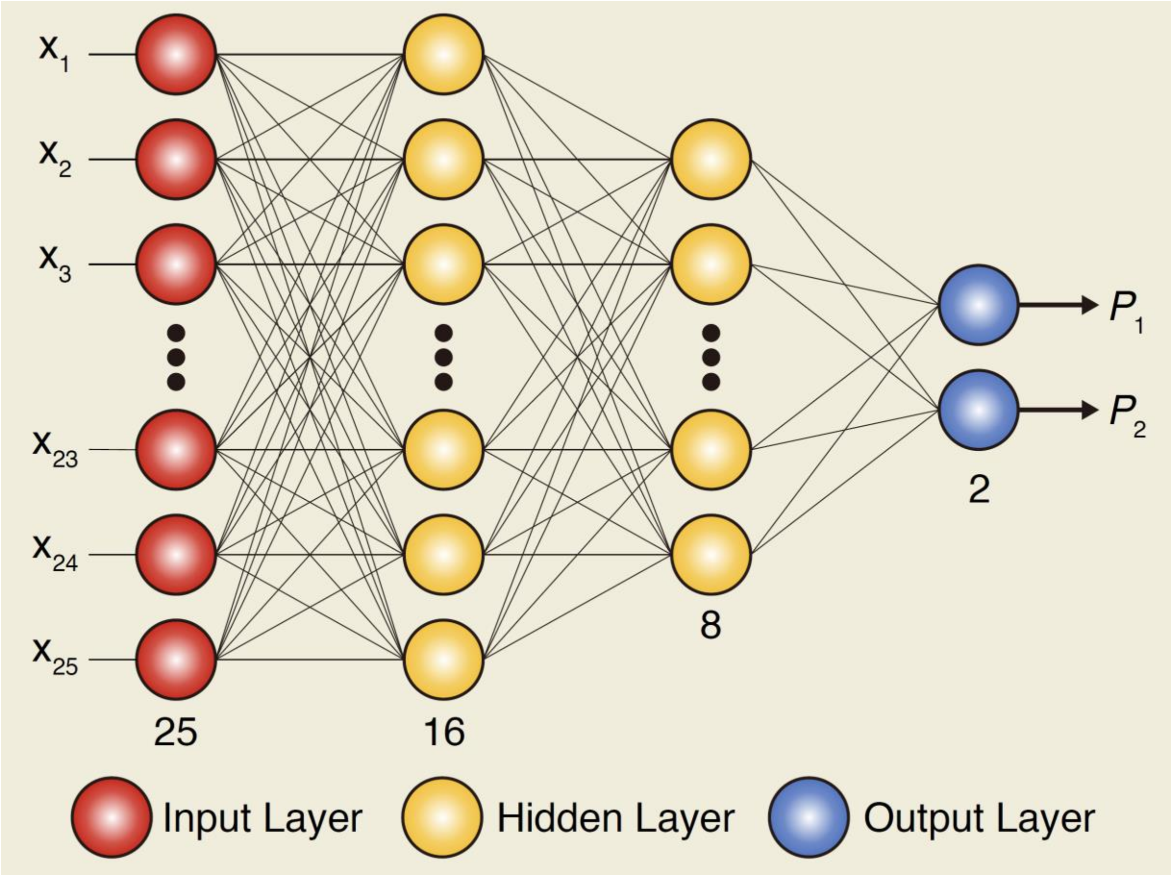

Fig. S6. Developing the DrABC Model through the Hierarchical Neural Network.

The prediction model was developed using the clinical characteristics in the discovery cohort and validated in the multi-center validation cohort. It starts with an input layer that contains 25 neurons (Table S2), which corresponds to features associated with the carriers of germline pathogenic variants in cancer predisposition genes. Then two hidden layers follows, with 16 and 8 neurons, respectively. Furthermore, a non-linear activation function, namely Scaled Exponential Linear Unit (SELU) (29), is attached to hidden layers' outputs, and it helps maintain the representation distributions to be close to unit Gaussian. Finally, we have an output layer with 2 neurons, which

comes with a sigmoid activation function, so that it produces two valid probabilities (i.e., in the range of [0,1]): P_1 and P_2.
