# Supplementary material for: DrABC: deep learning accurately predicts germline pathogenic mutation status in breast cancer patients based on phenotype data
Source: Genome Med. 2022 Feb 25;14:21. doi: 10.1186/s13073-022-01027-9 (PMC8876403; doi:10.1186/s13073-022-01027-9)
Supplement: Supplementary file 10 — Additional file 10: Figure S7. The Performance of the DrABC Model and Other Machine Learning Models Using an Inner Five-fold Cross-validation Strategy. [file 13073_2022_1027_MOESM10_ESM.pdf]

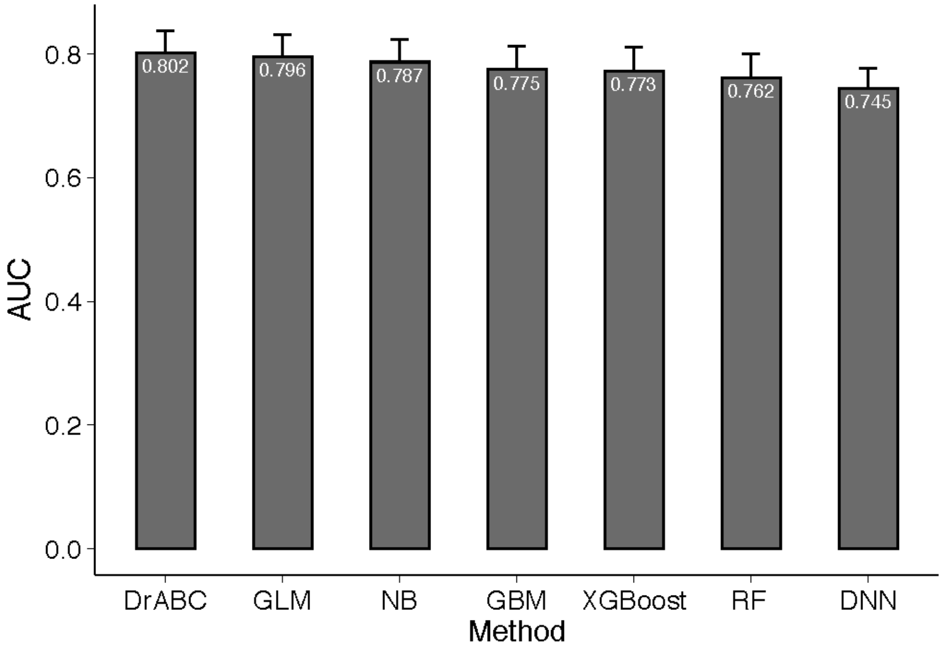

Fig. S7. The Performance of the DrABC Model and Other Machine Learning Models Using an Inner Five-fold Cross-validation Strategy.

To evaluate the difference between our hierarchical neural network model (DrABC) and other machine learning models, we explored six kinds of common machine learning algorithms, including a fixed grid of Generalized Linear Models (GLMs), a naive Bayes (NB) classifier, five pre-specified Gradient Boosting Machine (GBM) models, three pre-specified and a random grid of eXtreme Gradient Boosting (XGBoost) models, a default Random Forest (RF), a near-default Deep Neural Net (DNN) and a random grid of DNNs. All models were trained on the discovery dataset to predict whether a breast cancer patient carries germline pathogenic variants in any CPGs using an inner five-fold cross-validation strategy. For each algorithm family, we assessed and ranked models by AUCs and only the best model was retained to represent the maximum performance of each kind. As a result, DrABC achieved the optimal performance with an AUC of

0.802, which is slightly higher than the second-ranked algorithm family-GLM (AUC = 0.796). However, there was no significant difference between DrABC and the traditional machine learning models (all p>0.05) through the “DeLong’s test”.
