# Supplementary material for: DrABC: deep learning accurately predicts germline pathogenic mutation status in breast cancer patients based on phenotype data
Source: Genome Med. 2022 Feb 25;14:21. doi: 10.1186/s13073-022-01027-9 (PMC8876403; doi:10.1186/s13073-022-01027-9)
Supplement: Supplementary file 13 — Additional file 13: Figure S8. The Distribution of the Predicted Probabilities in Non-carriers and CPGs-carriers by the DrABC Model. [file 13073_2022_1027_MOESM13_ESM.pdf]

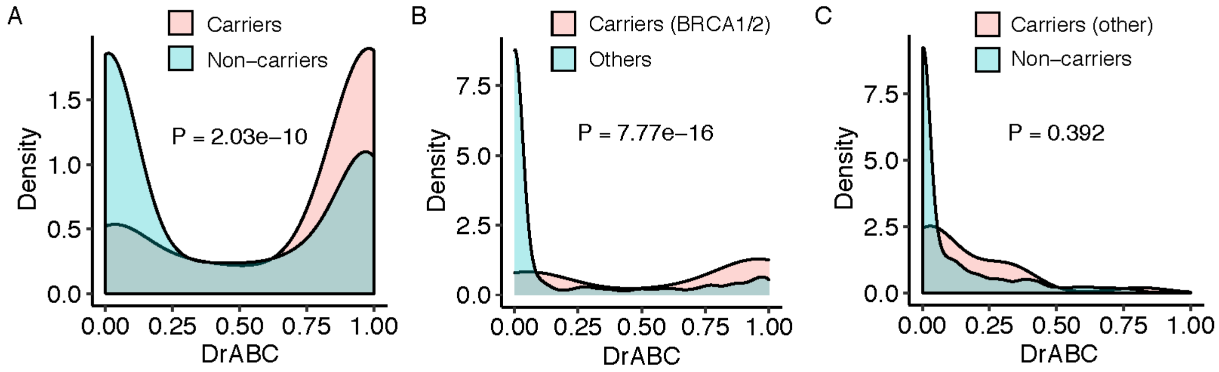

Fig. S8. The Distribution of the Predicted Probabilities in Non-carriers and CPGs-carriers by the DrABC Model.

A-B) The possibilities generated by DrABC were distributed differently between the non-carriers and the CPGs-carriers **(A)** or *BRCA1/2*-carriers **(B)**. **C)** However, they were indistinguishable between the non-carriers and the patients carrying GPVs in CPGs other than *BRCA1/2*.
