# Supplementary material for: DrABC: deep learning accurately predicts germline pathogenic mutation status in breast cancer patients based on phenotype data
Source: Genome Med. 2022 Feb 25;14:21. doi: 10.1186/s13073-022-01027-9 (PMC8876403; doi:10.1186/s13073-022-01027-9)
Supplement: Supplementary file 14 — Additional file 14: Figure S9. Performance of Risk Prediction Models for Breast Cancer Patients with germline Pathogenic Variants in Cancer Predisposition Genes other than BRCA1/2. [file 13073_2022_1027_MOESM14_ESM.pdf]

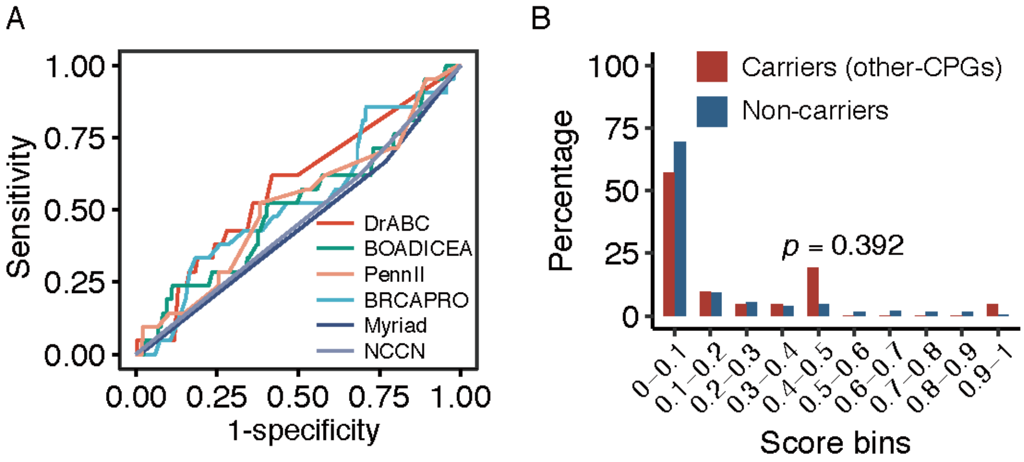

Fig. S9. Performance of Risk Prediction Models for Breast Cancer Patients with germline Pathogenic Variants in Cancer Predisposition Genes other than *BRCA1/2*.

A) The performance of DrABC was limited for germline pathogenic variants (GPVs) in any cancer predisposition genes (CPGs) other than *BRCA1/2*, with an AUC of 0.58, which was still higher than those for previous models (0.53 for BRCAPRO, 0.51 for BOADICEA, 0.44 for Myriad, 0.52 for PENNII, 0.47 for NCCN criteria). **B)** However, they were indistinguishable between non-carriers and patients carrying GPVs in CPGs other than *BRCA1/2*.
