# Supplementary material for: DrABC: deep learning accurately predicts germline pathogenic mutation status in breast cancer patients based on phenotype data
Source: Genome Med. 2022 Feb 25;14:21. doi: 10.1186/s13073-022-01027-9 (PMC8876403; doi:10.1186/s13073-022-01027-9)
Supplement: Supplementary file 15 — Additional file 15: Figure S10. The Contribution of Family Cancer History and Pathological Features to the DrABC Model. [file 13073_2022_1027_MOESM15_ESM.pdf]

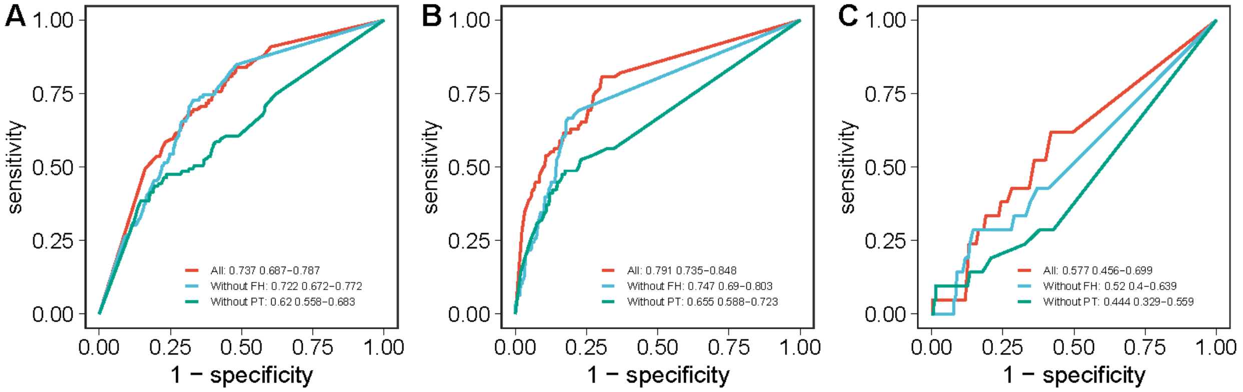

Fig. S10. The Contribution of Family Cancer History and Pathological Features to the DrABC Model.

When eliminating the family history data in the validation cohort, the performance of the DrABC model maintained with an AUC of 0.72 in predicting germline pathogenic variants (GPVs) in all cancer predisposition genes (CPGs) **(A)**, an AUC of 0.75 in predicting GPVs in *BRCA1/2* **(B)**, and an AUC of 0.52 in predicting GPVs in other CPGs **(C)**. However, when eliminating the data of pathological features in the validation cohort, the performance of the DrABC model dropped to AUC=0.62 in predicting GPVs in any CPGs **(A)**, AUC=0.66 in predicting GPVs in *BRCA1/2* **(B)**, and AUC=0.44 in predicting GPVs in other CPGs **(C)**.
