# Supplementary material for: DrABC: deep learning accurately predicts germline pathogenic mutation status in breast cancer patients based on phenotype data
Source: Genome Med. 2022 Feb 25;14:21. doi: 10.1186/s13073-022-01027-9 (PMC8876403; doi:10.1186/s13073-022-01027-9)
Supplement: Supplementary file 17 — Additional file 17: Figure S11. The Performance of Reconstructed Previous Models Which Were Trained in the Discovery Cohort and Tested in the Validation Cohort. [file 13073_2022_1027_MOESM17_ESM.pdf]

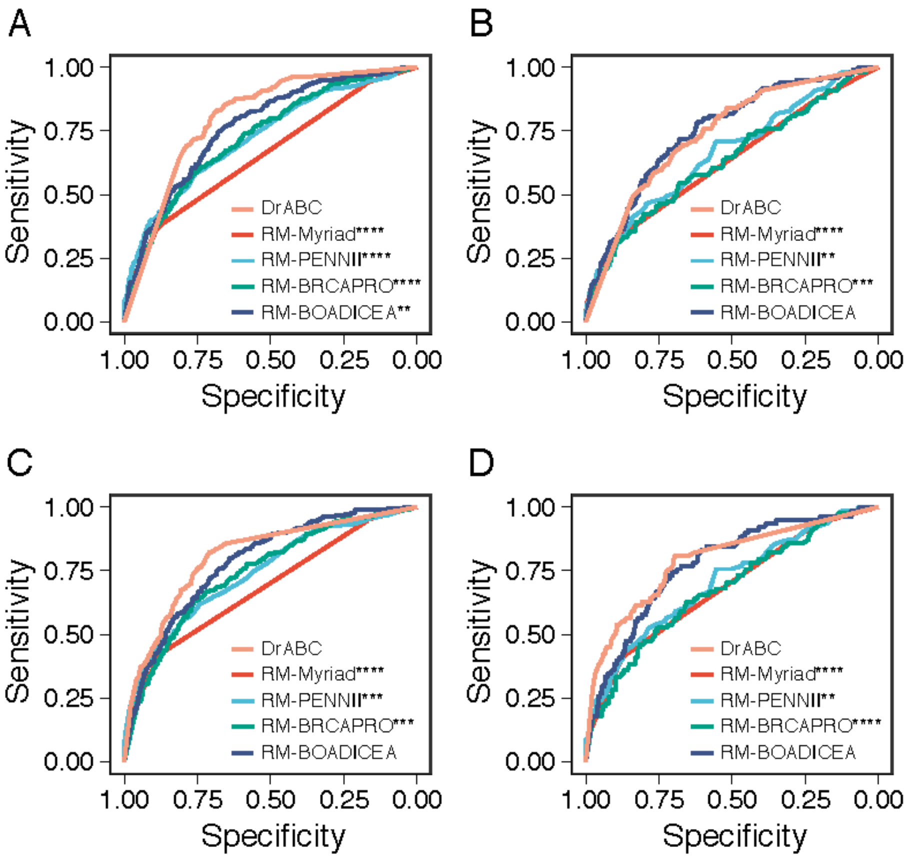

Fig. S11. The Performance of Reconstructed Previous Models Which Were Trained in the Discovery Cohort and Tested in the Validation Cohort.

A) In predicting GPVs in any CPG, DrABC was superior to the reconstructed models of BOADICEA, BRCAPRO, Myriad, and PENN II in the discovery cohort (p<0.01 when comparing each model with the DrABC; Table S6); **B)** DrABC was also superior to the reconstructed models of BRCAPRO, Myriad, and PENN II (p<0.01 when comparing each model with the DrABC; Table S6), but similar with the reconstructed BOADICEA model (p=0.48 when comparing each model with the DrABC; Table S6) in predicting GPVs in any CPG in the validation cohort. **C-D)** Similarly, in predicting GPVs in *BRCA1/2*, DrABC was superior to the reconstructed models of

BRCAPRO, Myriad, and PENN II in the discovery cohort **(C)** and the validation cohort **(D)** (p<0.01 when comparing each model with the DrABC; Table S6). However, there was no significant difference between the AUCs for DrABC and the reconstructed BOADICEA model in both the discovery cohort **(C)** and the validation cohort **(D)** (p=0.094 and 0.32 when comparing each model with the DrABC, respectively; Table S6). ** p<0.01, *** p<0.001, **** p<0.0001, when comparing with the DrABC. Abbreviation: DrABC, DNA-repair associated breast cancer; RM, reconstructed model.
