# Supplementary material for: DrABC: deep learning accurately predicts germline pathogenic mutation status in breast cancer patients based on phenotype data
Source: Genome Med. 2022 Feb 25;14:21. doi: 10.1186/s13073-022-01027-9 (PMC8876403; doi:10.1186/s13073-022-01027-9)
Supplement: Supplementary file 19 — Additional file 19: Figure S12. Online Website for the DrABC Model. [file 13073_2022_1027_MOESM19_ESM.pdf]

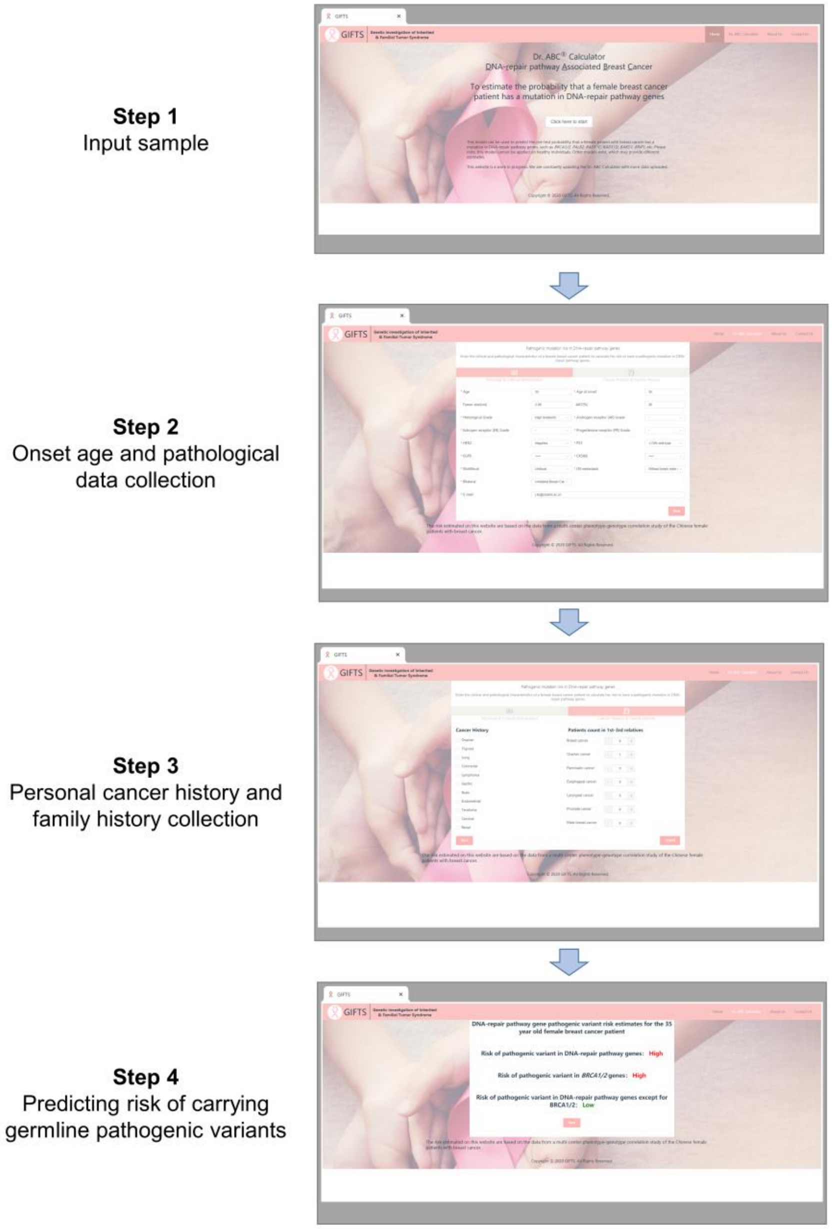

Fig. S12. Online Website for the DrABC Model.

A website interface (http://gifts.bio-data.cn/) was set up to accommodate extensions to the DrABC model and make it easily accessible to healthcare providers and researchers.
