# Supplementary material for: DrABC: deep learning accurately predicts germline pathogenic mutation status in breast cancer patients based on phenotype data
Source: Genome Med. 2022 Feb 25;14:21. doi: 10.1186/s13073-022-01027-9 (PMC8876403; doi:10.1186/s13073-022-01027-9)
Supplement: Supplementary file 20 — Additional file 20. A user guide for the DrABC model. [file 13073_2022_1027_MOESM20_ESM.pdf]

A user guide for the DrABC model

The DrABC (DNA-repair associated breast cancer) model was designed to calculate the risk of carrying a germline pathogenic variant in cancer predisposition genes for female patients with breast cancer. Currently, this model has only been trained and validated in the Chinese population, whose performance in other populations is still uncertain. The DrABC model was implemented in a website interface, which is easily accessible at http://gifts.bio-data.cn/. This online tool is available for healthcare providers and researchers. Please note this mode is not licensed to support clinical or diagnostic decisions.

Several steps to calculate the risk are described below.

1. Start calculation

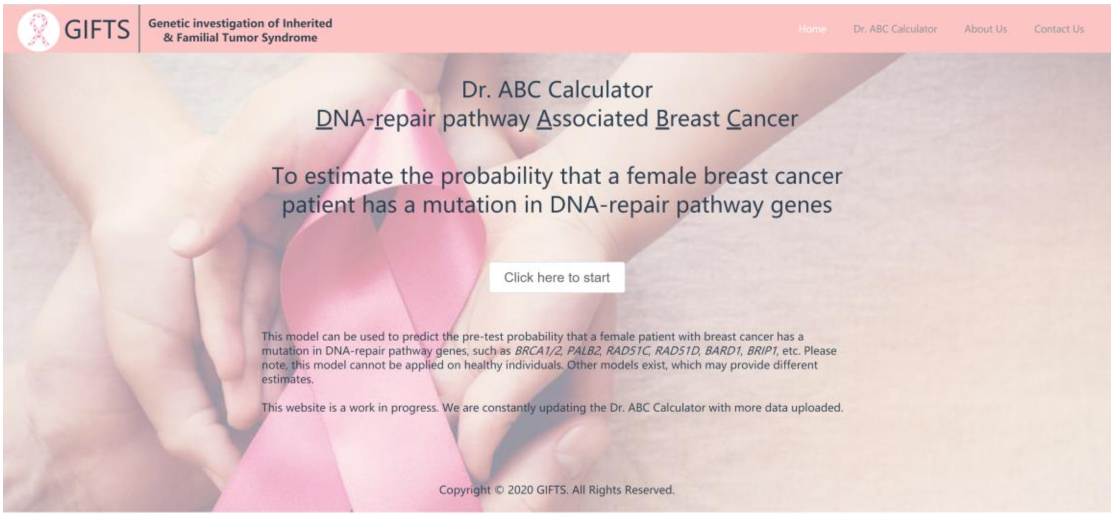

Click the [Click here to start] or the [Dr. ABC Calculator] button to start to calculate the risk of carrying a germline pathogenic variant in cancer

predisposition genes for female patients with breast cancer.

2. Provide the clinical information of the proband

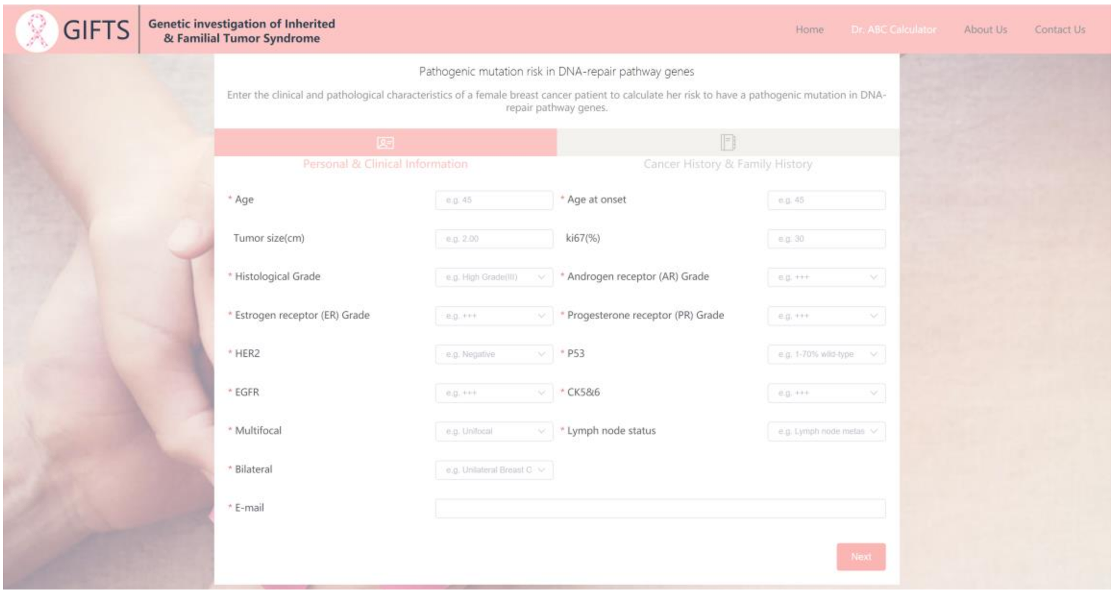

Information should be typed in the boxes as examples. Values with * are required, such as current age and the age at diagnosis. However, an “unknown” option was set for the pathological features. Thus, the missing data of the pathological features including Histological Grade, Androgen receptor (AR) Grade, Estrogen receptor (ER) Grade, Progesterone receptor (PR) Grade, HER2 status, P53 status, EGFR status, CK5&6, Bilateral or Unilateral, Multifocal or Unifocal, and Lymph node status, is tolerant. As numerical continuous variables and categorical variables are both present in our scenario, the missing data is imputed using proximity from random forest algorithm [Breiman L. Machine Learning 2001;45:5–32.]. An email of the researcher is also needed for further follow-up.

3. Provide the personal and family cancer history

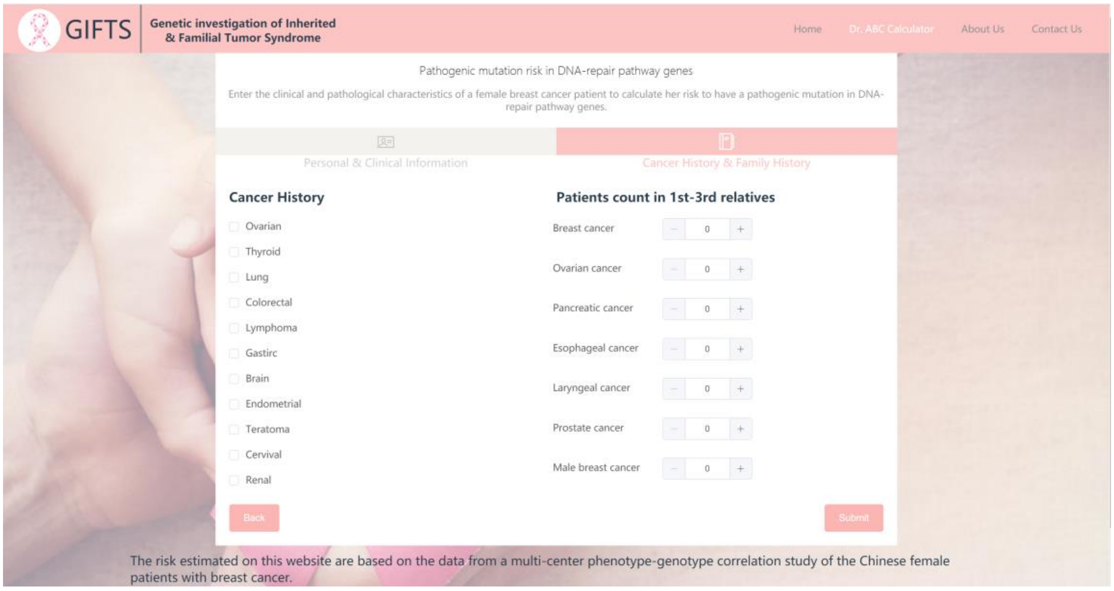

The user could select the cancer type which the proband has suffered. The patient number of the responding cancer type in the 1st-3rd degree relatives is also required. For example, if a sister, mother, anti, great-grandmother, second-cousins, and great-great-grandmother of a proband all have the breast cancer, the patients count in 1st-3rd relatives is 4 as the second-cousins and great-great-grandmother are the 4th-degree relatives. The ages at diagnosis of other cancer types for the proband and ages at diagnosis for the relatives are not required, as this information is often not available in clinical practice.

4. Risk estimation

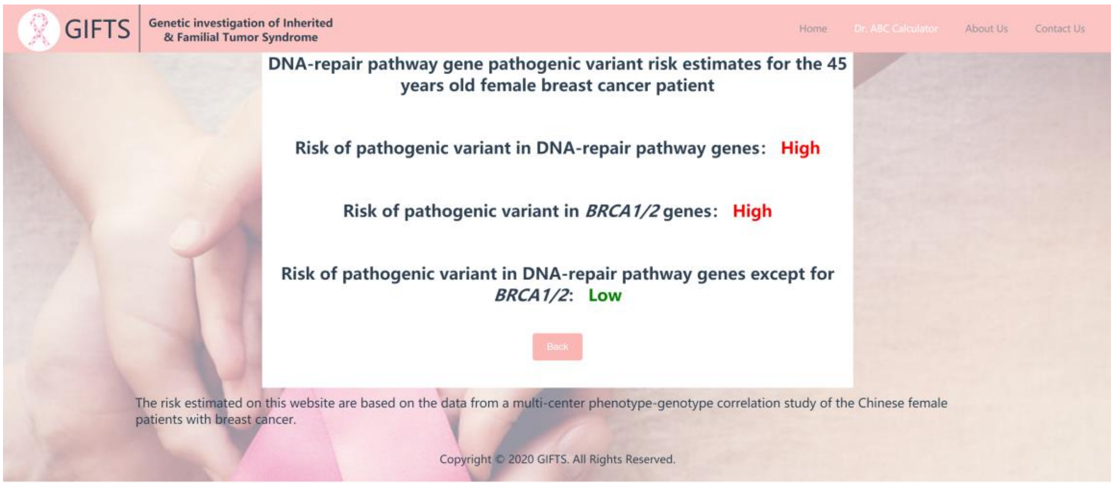

As a result, the DNA-repair pathway gene pathogenic variant risk for the female breast cancer patient with the responding features was estimated. The risks of carrying germline pathogenic variants in *BRCA1/2* or other cancer predisposition genes are predicted simultaneously, as different clinical management would be applied according to the different mutated genes.
