# Supplementary material for: DrABC: deep learning accurately predicts germline pathogenic mutation status in breast cancer patients based on phenotype data
Source: Genome Med. 2022 Feb 25;14:21. doi: 10.1186/s13073-022-01027-9 (PMC8876403; doi:10.1186/s13073-022-01027-9)
Supplement: Supplementary file 21 — Additional file 21: Figure S13. The Suggested Pipeline of Genetic Testing for Women with Breast Cancer. [file 13073_2022_1027_MOESM21_ESM.pdf]

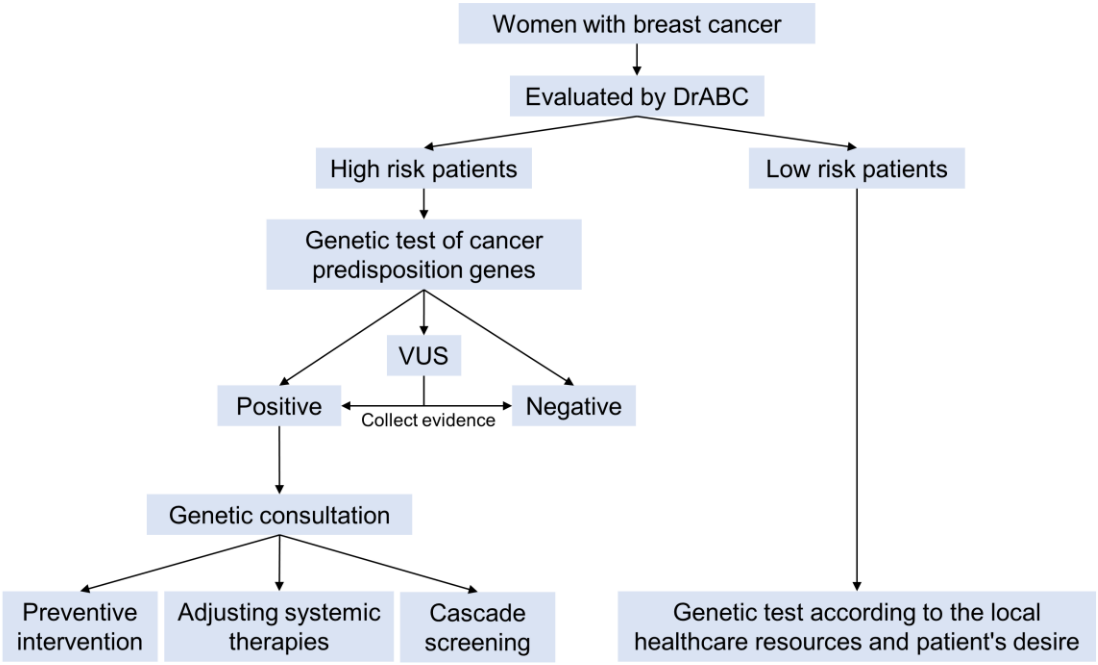

Fig. S13. The Suggested Pipeline of Genetic Testing for Women with Breast Cancer.

Based on the current guideline, we introduced an applicable pipeline for germline pathogenic variants (GPVs) carrier risk assessment of patients with breast cancer. The risk of carrying GPVs evaluated by DrABC is suggested to be performed prior to genetic testing. After the detection of GPVs, systemic therapy adjustment, preventive intervention, and cascade screening was recommended. This approach would balance between identifying more mutation carriers and testing fewer breast cancer patients. It will facilitate national guidelines on genetic consulting, preventive surgery, and reducing costs in healthcare for the mutation carriers. Testing the breast cancer patients with low GPVs risk would further increase the detection rate but should be decided according to the local healthcare resources and patient's desire.
